# Histamine activates an intracellular Ca^2+^ signal in normal human lung fibroblast WI-38 cells

**DOI:** 10.3389/fcell.2022.991659

**Published:** 2022-09-02

**Authors:** Roberto Berra-Romani, Ajelet Vargaz-Guadarrama, Josué Sánchez-Gómez, Nayeli Coyotl-Santiago, Efraín Hernández-Arambide, José Everardo Avelino-Cruz, Mario García-Carrasco, Monica Savio, Giorgia Pellavio, Umberto Laforenza, Alfredo Lagunas-Martínez, Francesco Moccia

**Affiliations:** ^1^ Department of Biomedicine, School of Medicine, Benemérita Universidad Autónoma de Puebla, Puebla, México; ^2^ Laboratory of Molecular Cardiology, Institute of Physiology, Benemérita Universidad Autónoma de Puebla, Puebla, México; ^3^ Department of Immunology, School of Medicine, Benemérita Universidad Autónoma de Puebla, Puebla, México; ^4^ Department of Molecular Medicine, University of Pavia, Pavia, Italy; ^5^ Direction of Chronic Infections and Cancer, Research Center in Infection Diseases, National Institute of Public Health, Morelos, México; ^6^ Laboratory of General Physiology, Department of Biology and Biotechnology “Lazzaro Spallanzani”, University of Pavia, Pavia, Italy

**Keywords:** histamine, intracellular Ca^2+^, lung fibroblasts, WI-38, Ca^2+^ oscillations, InsP_3_ receptors, store-operated Ca^2+^ entry

## Abstract

Histamine is an inflammatory mediator that can be released from mast cells to induce airway remodeling and cause persistent airflow limitation in asthma. In addition to stimulating airway smooth muscle cell constriction and hyperplasia, histamine promotes pulmonary remodeling by inducing fibroblast proliferation, contraction, and migration. It has long been known that histamine receptor 1 (H1R) mediates the effects of histamine on human pulmonary fibroblasts through an increase in intracellular Ca^2+^ concentration ([Ca^2+^]_i_), but the underlying signaling mechanisms are still unknown. Herein, we exploited single-cell Ca^2+^ imaging to assess the signal transduction pathways whereby histamine generates intracellular Ca^2+^ signals in the human fetal lung fibroblast cell line, WI-38. WI-38 fibroblasts were loaded with the Ca^2+^-sensitive fluorophore, FURA-2/AM, and challenged with histamine in the absence and presence of specific pharmacological inhibitors to dissect the Ca^2+^ release/entry pathways responsible for the onset of the Ca^2+^ response. Histamine elicited complex intracellular Ca^2+^ signatures in WI-38 fibroblasts throughout a concentration range spanning between 1 µM and 1 mM. In accord, the Ca^2+^ response to histamine adopted four main temporal patterns, which were, respectively, termed peak, peak-oscillations, peak-plateau-oscillations, and peak-plateau. Histamine-evoked intracellular Ca^2+^ signals were abolished by pyrilamine, which selectively blocks H1R, and significantly reduced by ranitidine, which selectively inhibits H2R. Conversely, the pharmacological blockade of H3R and H4R did not affect the complex increase in [Ca^2+^]_i_ evoked by histamine in WI-38 fibroblasts. In agreement with these findings, histamine-induced intracellular Ca^2+^ signals were initiated by intracellular Ca^2+^ release from the endoplasmic reticulum through inositol-1,4,5-trisphosphate (InsP_3_) receptors (InsP_3_R) and sustained by store-operated Ca^2+^ channels (SOCs). Conversely, L-type voltage-operated Ca^2+^ channels did not support histamine-induced extracellular Ca^2+^ entry. A preliminary transcriptomic analysis confirmed that WI-38 human lung fibroblasts express all the three InsP_3_R isoforms as well as STIM2 and Orai3, which represent the molecular components of SOCs. The pharmacological blockade of InsP_3_ and SOC, therefore, could represent an alternative strategy to prevent the pernicious effects of histamine on lung fibroblasts in asthmatic patients.

## Introduction

Asthma is a heterogeneous disease, generally characterized by chronic inflammation of the airways, defined by a clinical history of respiratory symptoms, such as wheezing, shortness of breath, chest tightness, and cough that vary in intensity and frequency, along with variable expiratory airflow (GBD Diseases and Injuries Collaborators, 2020). About 300 million people suffer from asthma worldwide and it is likely that, by 2025, another 100 million people will be affected by this disease (Dharmage et al., 2019).

The complex network of inflammatory responses in the pathophysiology of asthma involves the release of inflammatory mediators, such as cytokines, chemokines, proteases, and histamine (Murdoch and Lloyd, 2010). Histamine has been a widely recognized inflammatory mediator released from mast cells and could play a key role in the pathophysiology of asthma (Yamauchi and Ogasawara, 2019). Tomioka et al. (1984) estimated that the number of mast cells and the concentration of histamine in bronchoalveolar lavage fluid of asthmatic patients was higher than that of healthy subjects. In addition, Carroll et al. (2002) demonstrated that mast cell degranulation is related to the severity of asthma. Salomonsson et al. (2019) recently reported that elevated levels of circulating mast cell progenitors are related to reduced lung function in asthmatic patients.

*In vitro* studies demonstrated that histamine stimulated lung fibroblast collagen synthesis (Garbuzenko et al., 2004; Veerappan et al., 2013), migration (Kohyama et al., 2010), proliferation (Jordana et al., 1988; Veerappan et al., 2013) and human lung myofibroblast contraction (Horie et al., 2014). However, the transduction mechanisms whereby histamine leads to these effects in lung fibroblasts are still unclear. Histamine is thought to act by stimulating one or more of four types of plasmalemmal histaminergic receptor (HR) (H1R, H2R, H3R, and H4R) (Panula et al., 2015; Panula, 2021). The expression of H1R and, in less proportion, H2R has been reported in normal human lung fibroblasts (Veerappan et al., 2013) and their participation in promoting lung fibroblast activation has been proposed (Jordana et al., 1988; Garbuzenko et al., 2004; Kunzmann et al., 2007; Veerappan et al., 2013). In addition, H4R could promote lung fibroblast migration (Kohyama et al., 2010). Stimulation of H1R results in an increase in intracellular Ca^2+^ concentration ([Ca^2+^]_i_) in human valvular myofibrobasts (Liang et al., 2003), pteryglial derived fibroblasts (Maini et al., 2002), human gingival fibroblasts (Niisato et al., 1996; Ogata et al., 1999; Gutiérrez-Venegas and Rodríguez-Pérez, 2012), rheumatoid synovial fibroblasts (Zenmyo et al., 1995) human skin fibroblasts (Johnson et al., 1990), human subcutaneous fibroblasts (Pinheiro et al., 2013), and human lung fibroblasts (Zheng et al., 1994; Horie et al., 2014). While H3R are predominantly located in neurons (Abdulrazzaq et al., 2022), H4R are preferentially expressed in cells of the immune system and in blood forming organs, especially in mast cells, dendritic cells, basophils, eosinophils, monocytes, and T lymphocytes (Sarasola et al., 2021). However, immunostaining demonstrated that also human dermal fibroblasts express the H4R (Ikawa et al., 2008). Signaling mechanisms for the H4R receptor are much less well understood but appear to involve an increase in [Ca^2+^]_i_ (Panula et al., 2015).

An elevation in [Ca^2+^]_i_ in fibroblasts is key to protein synthesis, transcription factor activation, migration, progression through the cell cycle, and cell viability (Janssen et al., 2015). Resting [Ca^2+^]_i_ is maintained at very low levels (∼100–200 nM), while the extracellular Ca^2+^ concentration is 1000-fold higher (> 1 mM) and the Ca^2+^ concentration in the primary intracellular Ca^2+^ store, the endoplasmic reticulum (ER), fluctuates between 100–800 µM (Sadras et al., 2021a). An array of agonists, such as bradykinin, thrombin, trypsin, adenosine trisphosphate, angiotensin II, and histamine, increases the [Ca^2+^]_i_ in pulmonary fibroblasts at concentrations ranging from approximately 100 nM to 0.1 mM (Janssen et al., 2015). The [Ca^2+^]_i_ can be increased by two main mechanisms upon cellular stimulation: the first one is through the release of Ca^2+^ from the ER and the second one is through the inflow of Ca^2+^ from the extracellular medium. In several cell types, histamine bind to the G_q_-protein-coupled receptor (G_q_PCR), H1R, which activates phospholipase C (PLC). PLC hydrolyzes a membrane phospholipid, phosphatidylinositol 4, 5 bisphosphate (PIP_2_), to produce inositol-1, 4, 5-trisphosphate (InsP_3_). InsP_3_ triggers Ca^2+^ mobilization from the ER through InsP_3_ receptors (InsP_3_R) that are located on the ER membrane and can in turn activate adjacent ryanodine receptors (RyR) through the process of Ca^2+^-induced Ca^2+^ release (CICR) (Paltauf-Doburzynska et al., 2000; Clapham, 2007; Horie et al., 2014; Ishida et al., 2014; Berra-Romani et al., 2020). Extracellular Ca^2+^ can permeate the plasma membrane through a wide variety of ion channels, including voltage-operated channels (VOC) (Janssen et al., 2015; Rahman et al., 2016) and agonist-operated channels, which comprise three types of channels: 1) receptor-operated channels (ROC) (Saliba et al., 2015), which are ionotropic receptors stimulated by direct ligand binding, 2) second messenger-operated channels (SMOC), which are activated by intracellularly generated mediators, such as cyclic nucleotides, diacylglycerol (Hofmann et al., 2017), and arachidonic acid, and 3) store-operated Ca^2+^ channels (SOC), which are the main Ca^2+^ entry pathway in non-excitable cells. In the ER, Stromal Interaction Molecules (STIM1/2) act as sensors of ER Ca^2+^ concentration that, after a reduction in intraluminal Ca^2+^, multimerize and translocate towards peripheral ER cisternae to functionally interact with the Ca^2+^ permeable Orai channels on the plasma membrane (Bendiks et al., 2020). The following influx of Ca^2+^ has been termed store-operated Ca^2+^ entry (SOCE) and mediates agonist-induced Ca^2+^ influx in human fibroblasts isolated from several tissues, including lungs (Guzmán-Silva et al., 2015; Vazquez-de-Lara et al., 2018).

It has been demonstrated that histamine triggers an increase in [Ca^2+^]_i_ also in human lung fibroblasts (Zheng et al., 1994; Horie et al., 2014). However, the molecular mechanisms implicated in this response, which are likely to regulate the multiple fibroblast function involved in asthma remodeling, remain to be elucidated. Therefore, this study aimed to examine for the first time the mechanisms underlying histamine-induced increase in [Ca^2+^]_i_ in fetal human pulmonary WI-38 fibroblasts.

## Materials and methods

### Cell culture

Human fetal lung fibroblast cell lines were purchased from American Type Culture Collection, Collection WI-38 (ATCC^®^ CCL-75™) and cultured to 75% confluence in DMEM (Dulbecco’s Modified Eagle Medium) culture medium supplemented with 10% fetal bovine serum and 1% penicillin-streptomycin at 37°C in an atmosphere of 95% O_2_ and 5% CO_2_. Fibroblasts from passages 5–10 were seeded on coverslips for 24 h and then incubated for 48 h in medium devoid of serum.

### Physiological solutions

Physiological saline solution (PSS) with the following composition (in mM) 150 NaCl, 6 KCL, 1.5 CaCl2 1 MgCl2, 10 glucose, 10 HEPES was used for this study. To obtain Ca^2+^-free physiological saline solution (0Ca^2+^), Ca^2+^ was replaced with 2 mM NaCl and 0.5 mM EGTA was added as a Ca^2+^ chelator. Osmolarity was measured with an osmometer (Wescor 5500, Logan, UT, United States) solutions were adjusted to pH 7.4 with NaOH.

### Measurement of [Ca^2+^]_i_

The technique for Ca^2+^ measurement in fibroblasts has been previously described (Guzmán-Silva et al., 2015), and is explained in detail below. Fibroblasts attached to coverslips were washed twice PSS and incubated with 3 μM FURA-2 acetoxymethyl ester (FURA-2/AM) in PSS for 30 min at room temperature (21°C–23°C). Cells were incubated for 30 min in PSS free of FURA-2/AM. The coverslips were washed and fixed to the bottom of a Petri dish using a drop of silicone. The Petri dish was mounted on a stage of the Axiolab upright epifluorescence microscope (Carl Zeiss, Oberkochen, Germany), equipped with a 100 W mercury lamp (OSRAM HBO 50). A Zeiss X63 Achroplan objective (water immersion, working distance 2.0 mm, numerical aperture 0.9) was used to visualize fibroblasts. Cells were alternately excited at 340 nm and 380 nm using a filter spinning wheel with a shutter (Lambda 10, Sutter Instrument, Novato, CA, United States) and light emitted was detected at 510 nm. The Ca^2+^ signal was measured in individual fibroblasts, using software that allows to delimit each cell by drawing on the acquired images a region of interest. To control the camera (Extended-ISIS camera, Photonic Science, Millham, United Kingdom), the filter rotating wheel, as well as to draw the regions of interest of the fluorescent signal to be measured, a customized software, previously validated, running in LINUX environment, was used. The signal measurement was captured every 3 s and the images obtained were stored on a hard disk and subsequently converted into 340/380 ratio images using ImageJ software (National Institutes of Health, United States, https://imagej.nih.gov/ij/). An increase in 340/380 is indicative of an elevation in [Ca^2+^]_i_ (Ferrera et al., 2021; Remigante et al., 2021). Experiments were performed at room temperature (21°C–23°C). All experiments were performed in triplicate using 3 different passages of fibroblasts for each of the conditions.

### RT-qPCR

Total RNA was isolated from fibroblasts using QIAzol Lysis Reagent (Qiagen SpA, Milan, Italy), and reverse transcription was performed as described in (Ferrera et al., 2021; Negri et al., 2021). Reverse transcription was always performed in the presence (positive) or in the absence (negative control) of the reverse transcriptase enzyme (not shown), as shown elsewhere (Zuccolo et al., 2019; Zuccolini et al., 2022). cDNA amplification was performed using KAPA SYBR FAST qPCR Master Mix (KAPA BIOSYSTEMS, United States), and the primers used for amplification are listed the Table 1. The conditions were as follows: initial denaturation at 95°C for 5 min; 40 cycles of denaturation at 95°C for 10 s; annealing and extension at 60°C for 30 s, PCR products were separated on a 3% Nusieve^®^ (2:1) gel agarose, stained with ethidium bromide, and acquired with the iBrightTM CL1000 Imaging System (Thermo Fisher Scientific Inc., United States). The molecular weight of the PCR products was compared with the DNA molecular weight marker VIII (Roche Molecular Biochemicals, Italy).

**TABLE 1:** Primer sequences used for reverse transcription/polymerase chain reaction.

Gene	Primer sequences	Size (bp)	Accession number	
Orai1	Forward-5′-AGTTACTCCGAGGTGATGAG-3′	257	NM_032790.3	
	Reverse-5′-ATGCAGGTGCTGATCATGAG-3′			
Orai2	Forward-5′-CCATAAGGGCATGGATTACC-3′	334	NM_001126340.1	variant 1
	Reverse-5′-CAGGTTGTGGATGTTGCTCA-3′		NM_032831.2	variant 2
Orai3	Forward-5′-CCAAGCTCAAAGCTTCCAGCC-3′	159	NM_152,288.2	
	Reverse-5′-CAAAGAGGTGCACAGCCACCA-3′			
Stim1	Forward-5′-CCTCAGTATGAGGAGACCTT-3′	347	NM_003156.3	
	Reverse-5′-TCCTGAAGGTCATGCAGACT-3′			
Stim2	Forward-5′-AAACACAGCCATCTGCACAG-3′	186	NM_020860.2	
	Reverse-5′-GGGAAGTGTCGTTCCTTTGA -3′			
InsP_3_R1	Forward 5′-TCA​ACA​AAC​TGC​ACC​ACG​CT-3′	180	ENSG00000150995	
	Reverse 5′-CTC​TCA​TGG​CAT​TCT​TCT​CC-3′			
InsP_3_R2	Forward 5′-ACCTTGGG GTTAGTGGATGA-3′	158	ENSG00000123104	
	Reverse 5′-CCT​TGT​TTG​GCT​TGC​TTT​GC-3′			
InsP_3_R3	Forward 5′-TGG​CTT​CAT​CAG​CAC​TTT​GG-3′	173	ENSG00000096433	
	Reverse 5′-TGT​CCT​GCT​TAG​TCT​GCT​TG-3′			
RyR1	Hs00166991 Thermo Fisher Scientific	75	NM_000540.3	
RyR2	Hs00181461 Thermo Fisher Scientific	65	NM_001035	
RyR3	Hs00168821 Thermo Fisher Scientific	63	NM_001036.6	
Trpc1	Forward 5′-ATC​CTA​CAC​TGG​TGG​CAG​AA-3′	307		
	Reverse 5′-AAC​AAA​GCA​AAG​CAG​GTG​CC-3′			
Trpc3	Forward 5′-GGA​GAT​CTG​GAA​TCA​GCA​GA-3′	336	NM_001130698.1	v
	Reverse 5′-AAG​CAG​ACC​CAG​GAA​GAT​GA-3′		NM_003305.2	variant
Trpc4	Forward 5’-ACC​TGG​GAC​CTC​TGC​AAA​TA-3′	300	NM_016179.2	va
	Reverse 5′-ACA​TGG​TGG​CAC​CAA​CAA​AC-3′		NM_001135955.1	va
			NM_001135956.1	v
			NM_001135957.1	va
			NM_003306.1	va
			NM_001135958.1	va
Trpc5	Forward 5′-GAG​ATG​ACC​ACA​GTG​AAG​AG-3′	221	NM_012471.2	
	Reverse 5′-AGA​CAG​CAT​GGG​AAA​CAG​GA-3′			
Trpc6	Forward 5′-AGC​TGT​TCC​AGG​GCC​ATA​AA-3′	341	NM_004621.5	
	Reverse 5′-AAG​GAG​TTC​ATA​GCG​GAG​AC-3′			
Trpc7	Forward 5′-CAC​TTG​TGG​AAC​CTG​CTA​GA-3′	387	NM_020389.1	
	Reverse 5′-CAT​CCC​AAT​CAT​GAA​GGC​CA-3′			

### Data analysis

For the acquisition of fluorescence values, ImageJ software was used, and Origin Pro 2021 and GraphPad Prism 8.0 were used for graphing and statistical analysis of the results.

### Statistical design

Data were expressed as mean ± standard error (SE). Non-Gaussian data, identified by the D'Agostino and Pearson omnibus normality test (*p* ≤ 0.05) were statistically analyzed by the nonparametric Mann-Whitney test for two groups and Kruskal–Wallis for more than two groups. For normal data, an unpaired Student *t*-test for two groups and ANOVA for more than two groups were used. A value of *p* ≤ 0.05 was considered statistically significant.

Histamine concentration-response data were adjusted by the following Eq. 1:

$$Y=\frac{100}{1+\;\frac{EC_{50}}{\left[Histamine\right]}}$$ (1)

where *Y* is the response (relative to the Ca^2+^ transient amplitude), [Histamine] is the histamine concentration and the mean maximal effective concentration (*EC_50_*) is the [Histamine] that induced 50% of the maximal response.

## Results

### Histamine causes a heterogeneous Ca^2+^ signal in human lung fibroblasts of the WI-38 cell line

**FIGURE 1:**
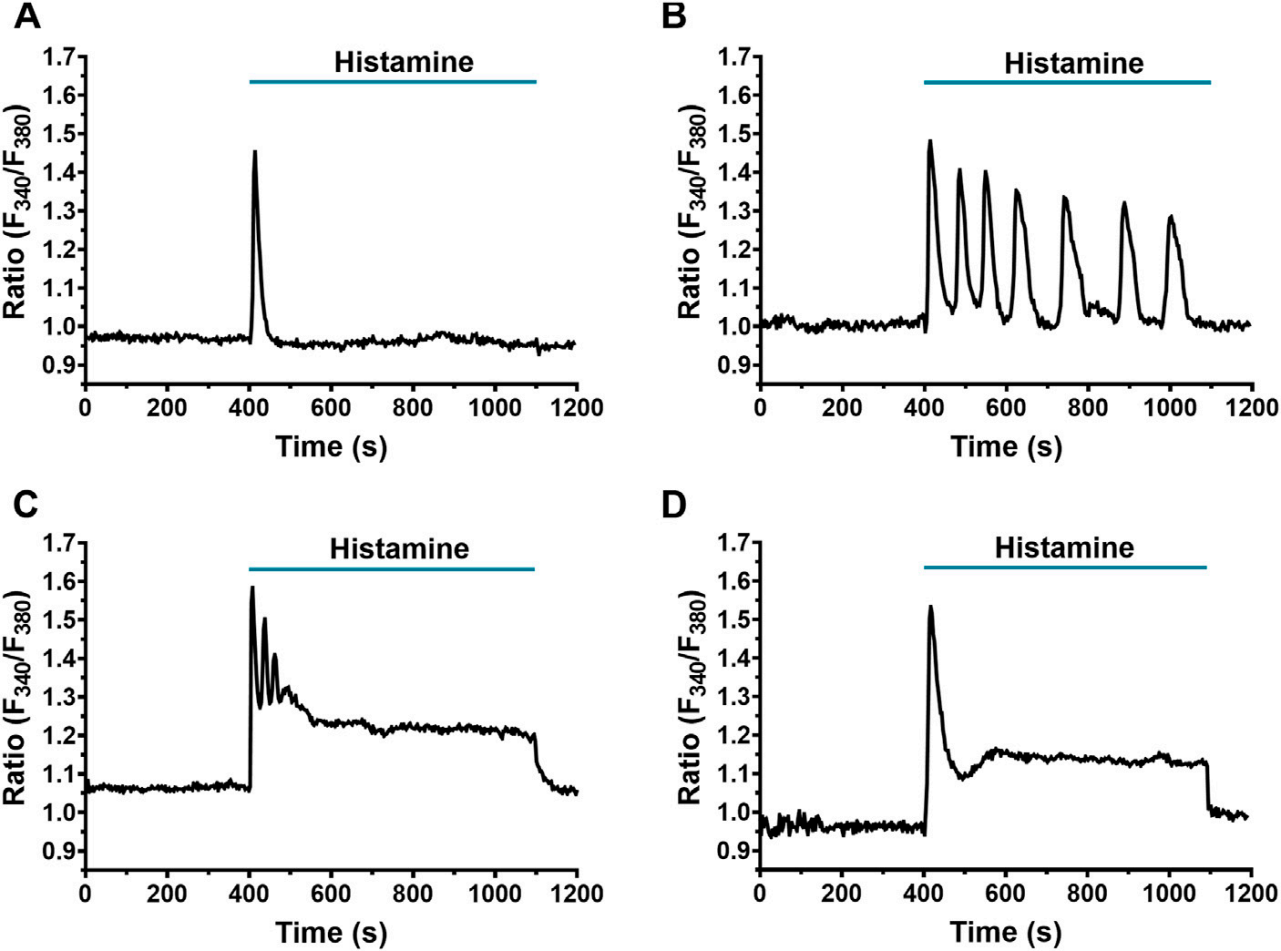
Heterogeneity in the Ca^2+^ response elicited by histamine in WI-38 human lung fibroblasts. Application of histamine (300 μM) elicited different Ca^2+^ signal patterns in FURA-2 AM-loaded WI-38 human lung fibroblasts. The intracellular Ca^2+^ signal consisted of **(A)** a rapid Ca^2+^ peak (spike) (19.28% of cells analyzed) which could be followed by **(B)** Ca^2+^ oscillations (64.70%) (peak-oscillations), **(C)** sustained plateau, superimposed by Ca^2+^ oscillations (13.39%) (peak-plateau-oscillations) or **(D)** only a plateau (0.65%) (peak-plateau). In this and the following figures, histamine was added at the time indicated by the horizontal bar drawn over the Ca^2+^ signal recording.

Using digital fluorescence imaging with FURA-2/AM [Ca^2+^]_i_ was measured simultaneously in several individual fibroblasts from the same population. A 22.5% of WI-38 fibroblasts displayed spontaneous Ca^2+^ oscillations, as also reported in human cardiac fibroblasts (Chen et al., 2010). These cells were, therefore, discarded from subsequent analysis (Faris et al., 2022). Application of histamine (300 μM), even in cells in the same microscopic field, elicited Ca^2+^ signals showing heterogeneous kinetics, which were classified into 4 different patterns: the first consisted of a rapid and transient increase in [Ca^2+^]_i_, termed peak (59/306 cells, 19.28%, Figure 1A **)**; the second in a peak followed by cyclic increases and decreases in [Ca^2+^]_i_, termed peak-oscillations (198/306 cells, 64.70%, Figure 1B); the third in a peak with oscillations and a sustained increase in [Ca^2+^]_i_, termed peak-plateau-oscillations (41/306 cells, 13.39%, Figure 1C); and finally, the fourth pattern which was the least frequent, consisted of a peak accompanied by a plateau, and was termed peak-plateau (2/306 cells, 0.65%, Figure 1D). Only 1.96% (6/306 cells) of the analyzed fibroblasts did not respond to histamine 300 μM.

### Histamine generates a Ca^2+^ signal in a concentration-dependent manner in WI-38 human lung fibroblasts

**FIGURE 2:**
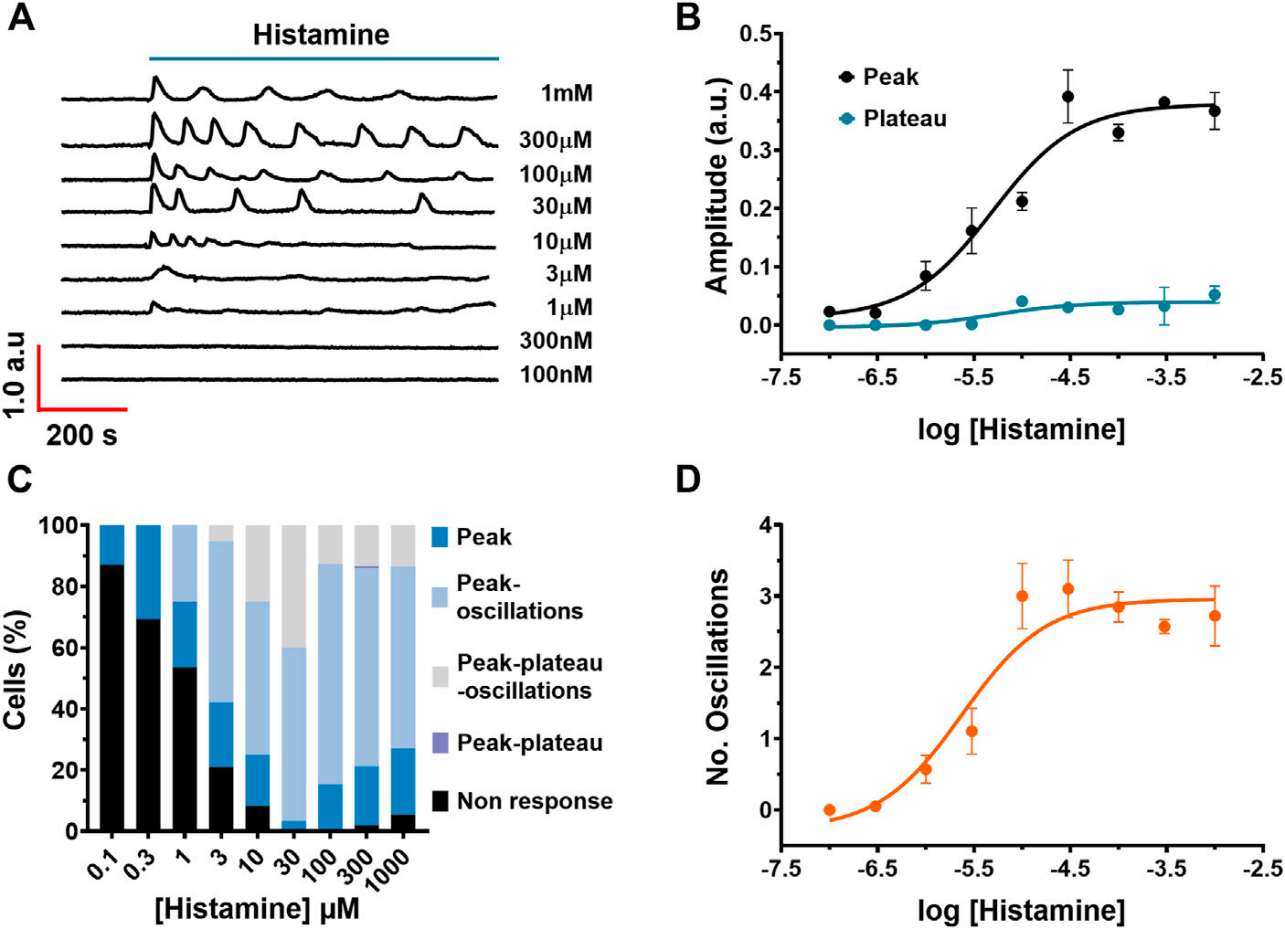
Concentration-dependent effect of histamine on Ca^2+^ signalling in WI-38 human lung fibroblasts. **(A)** Typical intracellular Ca^2+^ recordings in WI-38 cells loaded with FURA-2/AM exposed to different histamine concentrations ranging from 100 nM to 1 mM. The baseline of Ca^2+^ tracings has been shifted to avoid their overlapping for representation proposes. **(B)** Non-cumulative concentration-response relationship. Data points are the mean ± SE of the initial Ca^2+^ peak amplitude (black circles) or plateau amplitude (blue circles) plotted against the logarithm of histamine concentration. The continuous curves were obtained by fitting the data to Eq. 1, as shown in Materials and methods, which yielded EC_50_ values of 4.96 and 5.25 µM for peak amplitude (black line) and plateau amplitude (blue line), respectively. Data points were obtained of at least 19 cells. **(C)** Percentage of cells that presented each of the Ca^2+^ response patterns indicated in function of histamine concentrations applicated to WI-38 fibroblasts. **(D)** Data points are the mean ± SE of the number of oscillations measured over the first 400 s after histamine application, plotted against the logarithm of histamine concentration. The continuous curve was obtained by fitting the data to Eq. 1, as shown in Materials and methods, which yielded EC_50_ values of 2.38 µM.

Typical recordings of the Ca^2+^ signals evoked by different histamine concentrations (100 nM–1 mM) are shown in Figure 2A. Histamine did not elicit any discernible increase in [Ca^2+^]_i_ at very low concentrations, such as 100 and 300 nM. The Ca^2+^ response to histamine appeared at 1–3 µM; at these concentrations, the Ca^2+^ signal arising in most WI-38 cells displayed a single peak in response to agonist stimulation. However, at 10 µM histamine, the Ca^2+^ peak was followed by a short train of consecutive Ca^2+^ oscillations. The number of oscillations over 60 min of histamine application was increased at histamine concentrations ranging from 30 µM up to 1 mM. The non-cumulative concentration-response curve of histamine-induced elevation in [Ca^2+^]_i_ is depicted in Figure 2B (black circles), which shows that the increase in histamine concentration produces an increase in the amplitude of the initial Ca^2+^ response (peak). The maximum increase in the peak amplitude was observed at concentrations higher than 300 µM, whereas raising histamine concentration up to 1 mM did not significantly augment the magnitude of the response. Slight stimulation occurred at 3 µM, while no effect was detectable at concentrations lower than 1 µM (100 nM and 300 nM). The concentration of histamine required to produce a half maximal response (EC_50_), which was calculated by fitting the concentration-response curve as described in Materials and Methods, was 4.96 μM (Figure 2B, black circles). Likewise, in cells that presented a plateau in the Ca^2+^ waveform, the *EC_50_* of the plateau amplitude was equal to 5.25 μM (Figure 2B, blue circles). In order to assess whether the pattern of the Ca^2+^ signal was dependent on histamine concentration, the frequency of each Ca^2+^ signature detected at each histamine concentration (100 nM–1 mM) was calculated (Figure 2C). The data indicate that the spike pattern (see Figures 1A, 2C) is more common when fibroblasts are stimulated with low histamine concentrations (100 nM–1 µM), whereas the spike-oscillations patterns is more frequent as the histamine concentration is increased (3 μM–1 mM) (see Figures 1B,C, 2B). In accord, the number of oscillations recorded over the first 400 s after histamine application was increased in a histamine concentration-response manner with a EC_50_ = 2.37 µM (Figure 2D).

### Desensitization of the Ca^2+^ response by repeated stimulation of WI-38 human lung fibroblasts with histamine

**FIGURE 3:**
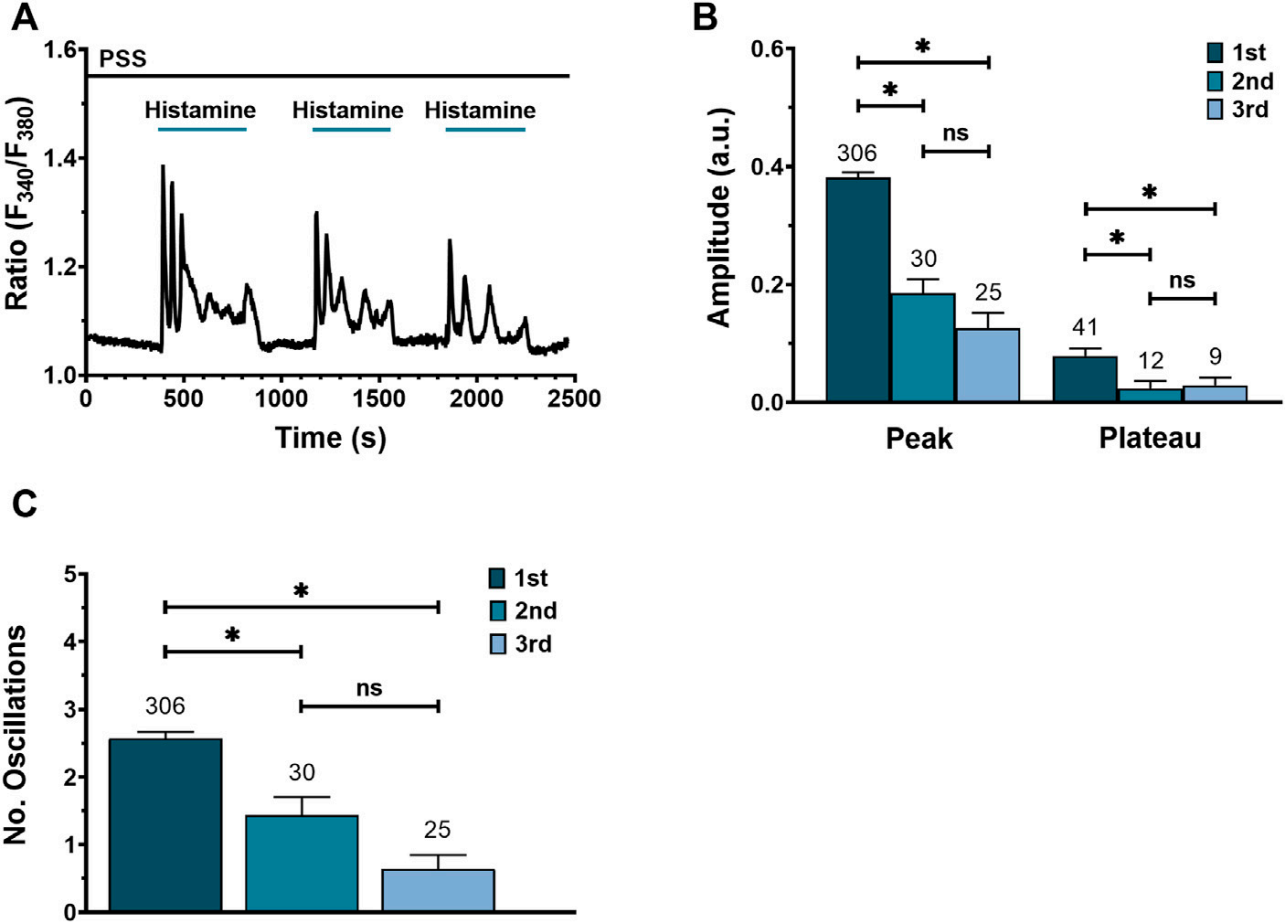
Effect of consecutive histamine applications on Ca^2+^ signal in WI-38 human lung fibroblasts WI-38. **(A)** Representative recording of a triple application of histamine (300 μM) in one cell, each followed by a washout with physiological saline solution (PSS). **(B)** Mean ± SE of peak and plateau amplitudes of the Ca^2+^ transient evoked by 3 consecutive histamine applications: first application (1st), second application (2nd), third application (3rd). **(C)** Mean ± SE of the number of oscillations measured over 400 s after each histamine application. The number in the figure represents the number of cells studied. Comparison between groups was performed using the Kruskal–Wallis test (* = *p* ≤ 0.05; ns = no statistically relevant differences between groups).

Homologous desensitization is a feature of GqPCRs, including H1R (Chen et al., 2014; Burghi et al., 2021). Figure 3A shows a typical Ca^2+^ recording from a WI-38 fibroblast exposed to three consecutive applications of 300 μM histamine followed by PSS washout. Histamine elicited a similar Ca^2+^ response consisting in an initial Ca^2+^ peak followed by sustained plateau, superimposed by Ca^2+^ oscillations. However, the peak and plateau amplitudes (Figure 3B), as well as the number of oscillations (Figure 3C), were significatively reduced by repetitive histamine stimulation**.** These data suggest that the application of a maximal concentration of histamine (300 μM) to the same fibroblast leads to receptor desensitization.

### Histamine-evoked elevation in [Ca^2+^]_i_ in WI-38 human lung fibroblasts is primarily mediated through activation of H1R and to a lesser extent through H2R

**FIGURE 4:**
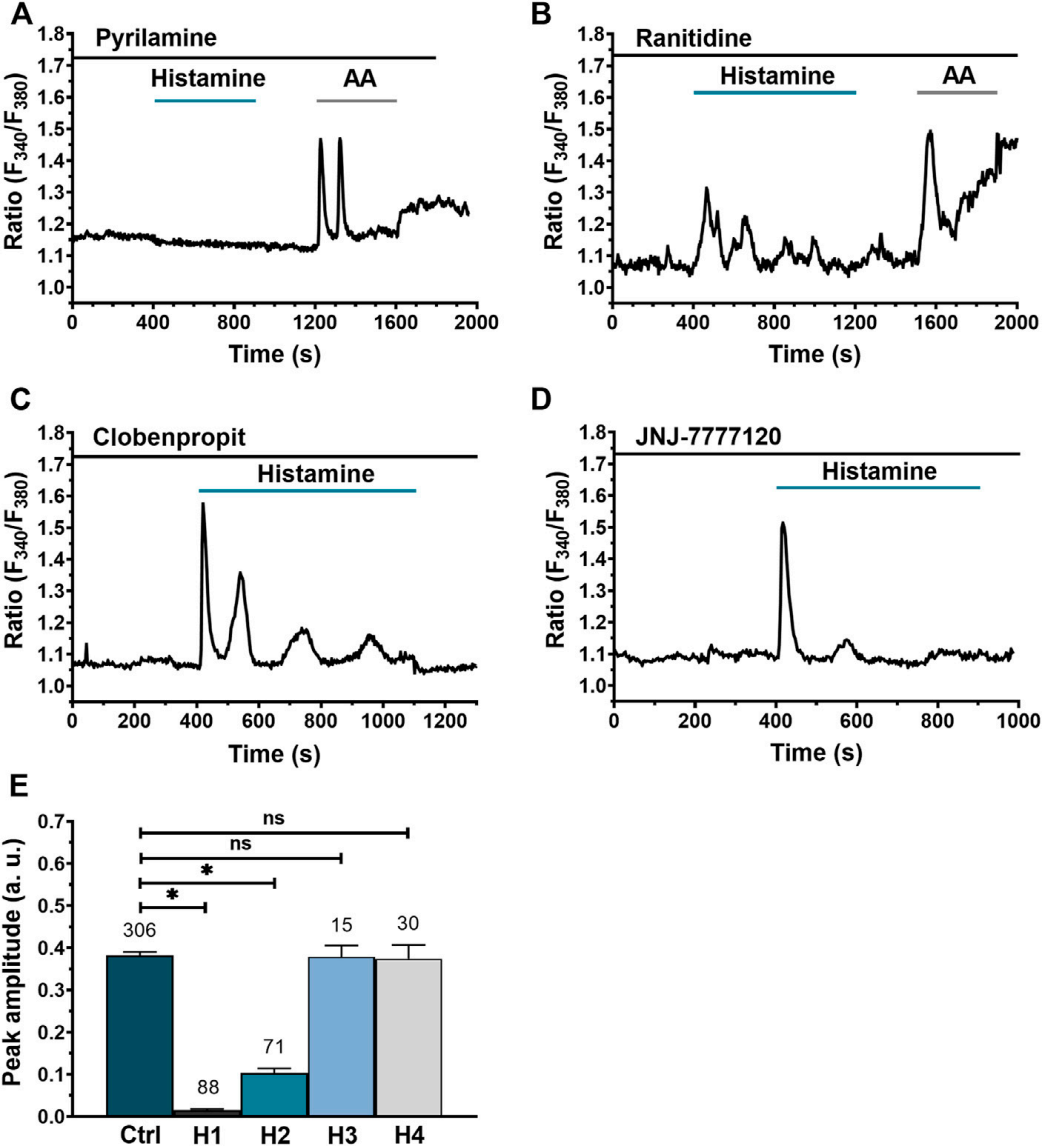
Dissection of histaminergic receptors (HR) responsible for histamine-evoked Ca^2+^ signal in WI-38 human lung fibroblasts. **(A)** Typical recording of histamine-evoked Ca^2+^ signal in the presence of the H1R antagonist, pyrilamine (100 μM). **(B)** Typical recording of the histamine-evoked Ca^2+^ signal in the presence of the H2R antagonist, ranitidine (50 μM). **(C)** Typical recording of the histamine-evoked Ca^2+^ signal in the presence of the H3R antagonist, clobenpropit (50 μM). **(D)** Typical recording of histamine-evoked Ca^2+^ signal in the presence of H4R antagonist, JNJ-7777120 (10 μM). All histaminergic antagonists were preincubated for 30 min prior to histamine application (incubation time not shown). For experiments showed in **(A,C)**, arachidonic acid (AA 50 μM) was applied after histamine application to corroborate cell viability. **(E)** Mean ± SE of the peak amplitude of the Ca^2+^ transient evoked by histamine (300 μM) in the absence (Ctrl) and presence of the HR antagonists: pyrilamine 100 μM (H1), ranitidine 50 μM (H2), clobenpropit 50 μM (H3) and JNJ-7777120 10 μM (H4). The numbers in the figure represents the number of cells studied. Comparison between groups was performed using the Kruskal–Wallis test (* = *p* ≤ 0.05) (ns = no statistically relevant differences between groups).

In order to elucidate the HR subtype through which histamine triggers an intracellular Ca^2+^ signal in WI-38 human lung fibroblasts, specific HR antagonists were used: for H1Rs pyrilamine (100 μM), for H2Rs ranitidine (50 μM), for H3R clobenpropit (50 μM) and, finally, for the H4R receptor, NJ7777120 (10 μM). After 30 min preincubation and in the continuous presence of the histaminergic antagonists, histamine (300 µM) was applied as indicated by the green bars. In order to confirm cell viability, arachidonic acid (AA) 50 µM was applied in cells in which histamine failed to induce an increase in [Ca^2+^]_i_ (Berra-Romani et al., 2019). Pharmacological manipulation of HR in WI-38 human lung fibroblasts revealed that H1R blockage completely abolished the histamine-evoked Ca^2+^ signal (Figure 4A), H2R blockage significantly decreased the amplitude of the Ca^2+^ signal (Figure 4B), while H3R (Figure 4C) and H4R blockage (Figure 4D) had no significant effect. In Figure 4E, the statistical comparison between the mean ± SE peak amplitude of the Ca^2+^ signals evoked by histamine 300 μM in the absence (Ctrl) and presence of the different antihistaminergic receptors antagonists (H1R, H2R, H3R, and H4R) are summarized. These results indicate that, for the [Ca^2+^]_i_ elevation to take place in the lung fibroblast cell line, WI-38, the activation of mainly H1R and, to a lesser extent, H2R is necessary, whereas H3R and H4R seem to play no role.

### Histamine-evoked Ca^2+^ signals in WI-38 lung fibroblasts do not involve Gα_i/o_ activation but require PLCβ recruitment and ER Ca^2+^ release

**FIGURE 5:**
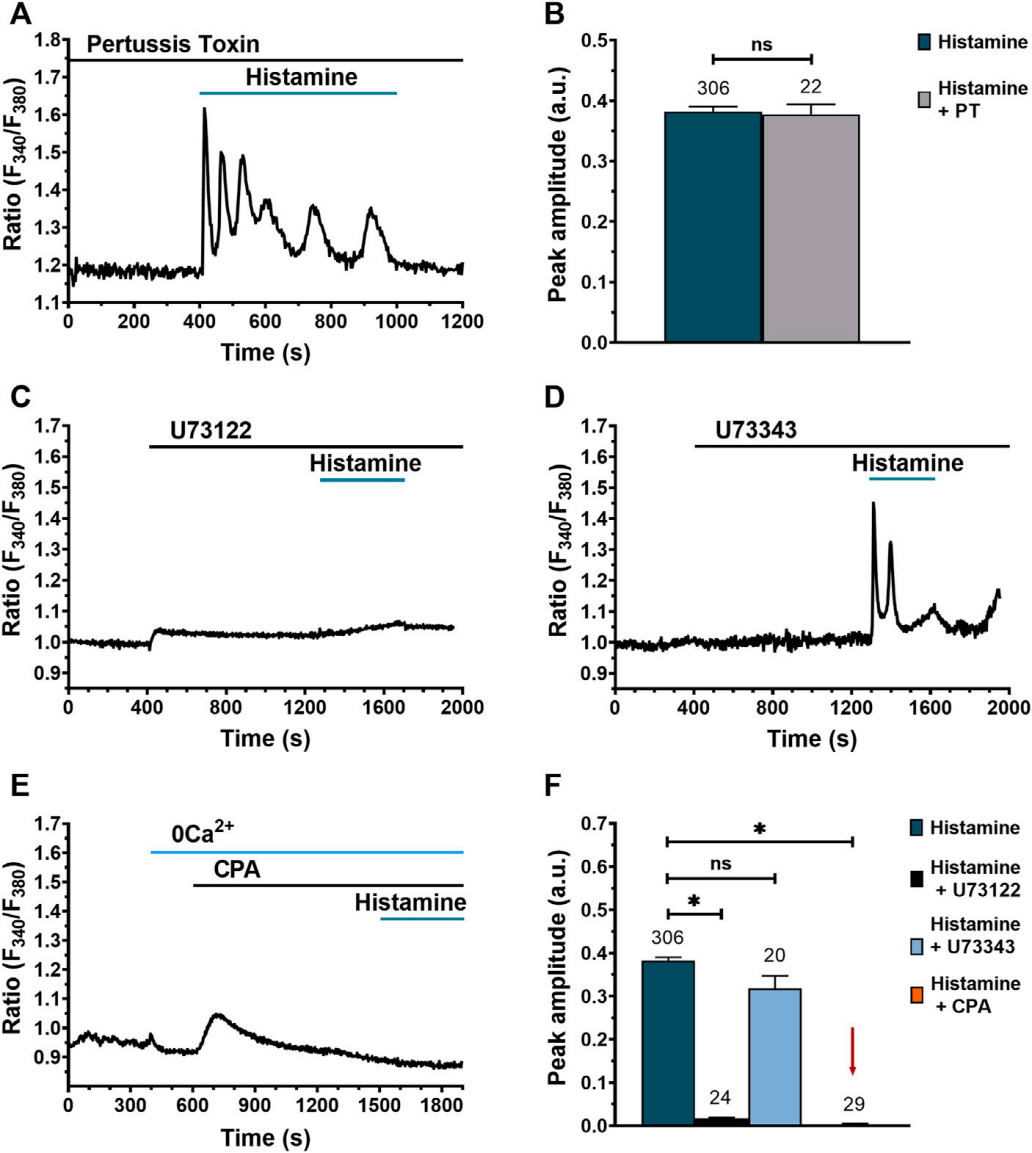
Histamine-evoked Ca^2+^ signals in WI-38 human lung fibroblasts do not involve Gα_i/o_ activation but require PLC and ER Ca^2+^ release. **(A)** Typical recording of the Ca^2+^ response to histamine (300 μM) in fibroblasts preincubated for 30 min with 100 ng/ml pertussis toxin (PT). **(B)** Mean ± SE of the peak amplitude of histamine-evoked Ca^2+^ transients in the absence (green bar) and presence of pertussis toxin (gray bar). Comparison between groups was performed using the Student’s t-test (ns = no statistically relevant differences between groups). **(C)** Typical recording of the effect of histamine (300 μM) on Ca^2+^ signal in cells pretreated for 15 min with U73122 (10 μM), a specific PLC inhibitor. **(D)** Typical recording of the Ca^2+^ response to histamine (300 μM) in cells pretreated for 15 min with U73343 (10 μM), an inactive analog of U73122. **(E)** Representative recording of the Ca^2+^ signal evoked by histamine (300 μM) in cells pretreated with CPA (10 μM) in the absence of extracellular Ca^2+^ (0Ca^2+^). **(F)** Mean ± SE of the peak Ca^2+^ response to histamine (300 μM; green bar) in the presence of U73122 10 μM (black bar), U73343 10 μM (blue bar) and CPA 10 μM (orange bar not visible, marked with a red arrow). The numbers in the figure represent the number of cells studied. Statistical comparison between groups was performed using ANOVA test (* = *p* ≤ 0.05).

The results shown in Figures 4C–E suggest that H3R and H4R do not participate in histamine-activated Ca^2+^ signaling. H3R and H4R are canonically coupled to Gα_i/o_ proteins (Haas et al., 2008). Therefore, a reliable strategy to corroborate the lack of H3R and H4R involvement in histamine response would be to demonstrate that pertussis toxin (PT), a selective inhibitor of Gα_i/o_ protein signalling, does not modify histamine-evoked Ca^2+^ signals. Indeed, as shown in Figure 5A, preincubation (30 min) with 100 ng/ml PT did not prevent histamine from increasing intracellular [Ca^2+^]_i_ in WI-38 fibroblasts. In accord, the amplitude of initial Ca^2+^ transient (peak) was not statistically different from untreated cells (Figure 5B). These results confirm that histamine-evoked increase in [Ca^2+^]_i_ in human lung WI-38 fibroblasts is insensitive to the inhibition of Gα_i/o_ by PT (Figures 5A,B). After confirming that H1R and H2R are involved in the Ca^2+^ response to histamine, we turned to dissect out the molecular underpinnings of the Ca^2+^ transient by using the following drugs: 1) U73122 (10 μM), a selective inhibitor of PLC (Moccia et al., 2006; Berra-Romani et al., 2012; Berra-Romani et al., 2020); 2) U73343 (10 μM), an inactive analogue of U73122 (Guzmán-Silva et al., 2015); and 3) cyclopiazonic acid (CPA) (10 μM), a selective inhibitor of sarco-edoplasmic reticulum calcium ATPase (SERCA) pump (Guzmán-Silva et al., 2015; Berra-Romani et al., 2020). The results obtained showed that PLC inhibition upon preincubation (15 min) with U73122 suppresses histamine-evoked Ca^2+^ signal (Figures 5C,F), whereas its inactive analog, U73343, does not affect the Ca^2+^ response (Figures 5D,F). Likewise, ER emptying *via* Ca^2+^ leak channels after SERCA inhibition with CPA in an extracellular Ca^2+^-free environment (0Ca^2+^) prevented the Ca^2+^ response to histamine (Figures 5E,F). These results indicate that the Ca^2+^ transient generated after histamine stimulation in WI-38 human lung fibroblasts is due to PLC activation and Ca^2+^ release from the ER.

### InsP_3_R play an important role in histamine-evoked Ca^2+^ signaling in WI-38 human lung fibroblasts

**FIGURE 6:**
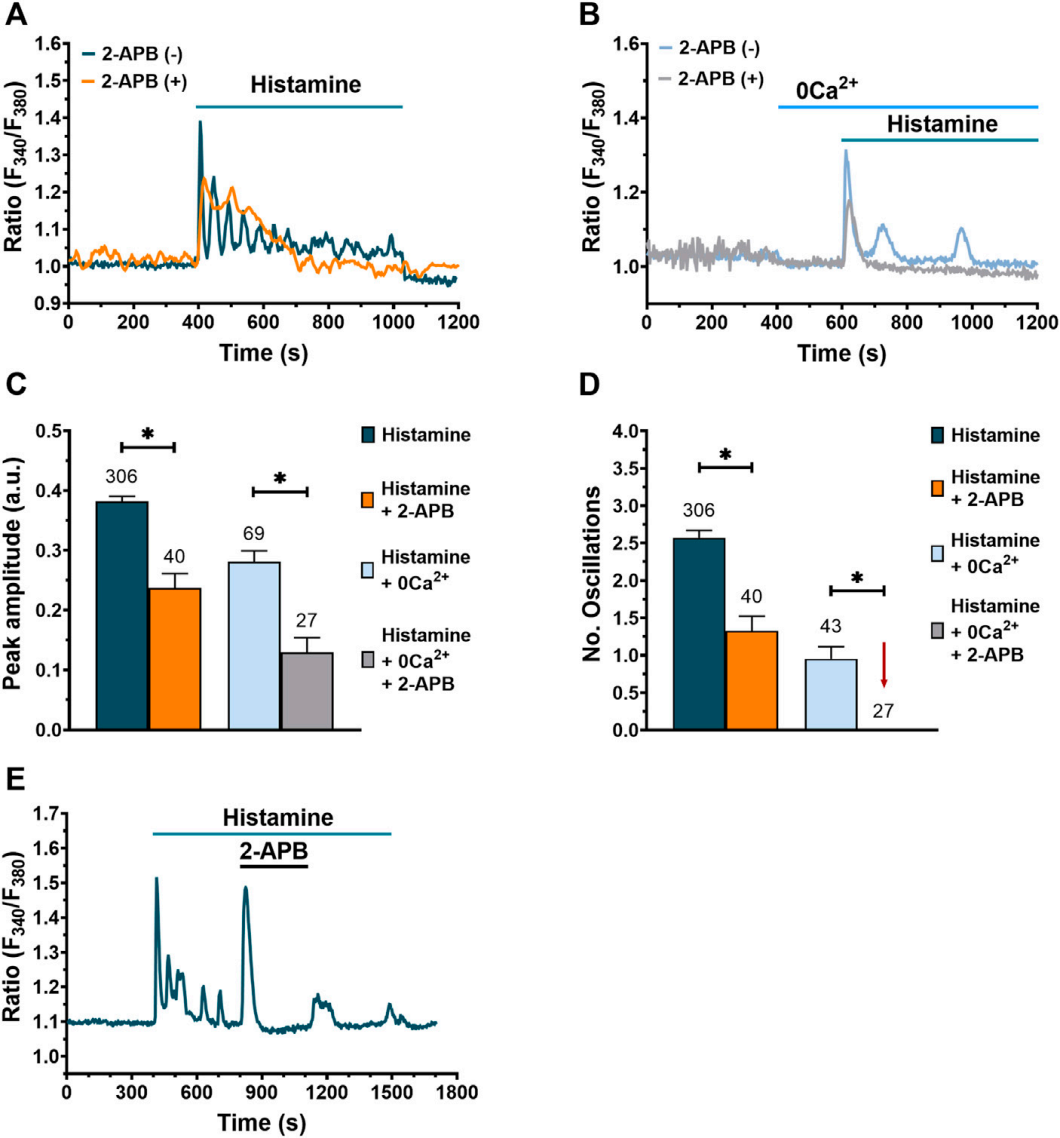
Involvement of InsP_3_R in histamine-evoked Ca^2+^ signals in WI-38 human lung fibroblasts. **(A)** Typical recording of the Ca^2+^ response to histamine (300 µM) in fibroblasts pre-incubated with 2-APB (50 μM) for 20 min [orange trace, 2-APB (+)] and its control [dark green trace, 2-APB (-)]. **(B)** Typical recording of histamine-evoked Ca^2+^ signal in absence of extracellular Ca^2+^ (0Ca^2+^) after pre-incubation of fibroblasts for 20 min with 2-APB (50 μM) [gray trace, 2-APB (+)] and its control [blue trace, 2-APB (-)]. In **(A**,**B**) basal Ca^2+^ levels were aligned for comparative purposes. **(C)** Mean ± SE of the peak amplitude of the Ca^2+^ response to histamine (300 µM) in normal extracellular Ca^2+^ and in absence (dark green bar) or presence of 50 μM 2-APB (orange bar). Mean ± SE of the peak amplitude of the Ca^2+^ response to histamine (300 µM) in absence of extracellular Ca^2+^ (0Ca^2+^) and in absence (blue bar) or presence of 50 μM 2-APB (gray bar). Statistic comparison between groups was performed using Mann-Whitney and t-Student test respectively (* = *p* ≤ 0.05). **(D)** Mean ± SE of the number of oscillations evoked by histamine (300 µM) in normal extracellular Ca^2+^ and in absence (dark green bar) or presence of 50 μM 2-APB (orange bar). Mean ± SE of the number of oscillations evoked by histamine (300 µM) in the absence of extracellular Ca^2+^ (0Ca^2+^) and in absence (blue bar) or presence of 50 μM 2-APB (gray bar, gray bar not visible, marked with a red arrow). Statistical comparison between groups was performed using Mann-Whitney and *t*-Student test respectively (* = *p* ≤ 0.05). The numbers in the figure represents the number of cells studied. **(E)** Typical recording of the Ca^2+^ signal evoked by histamine and the effect of 2-APB application.

Having demonstrated that the ER Ca^2+^ stores contributes to histamine-evoked Ca^2+^ signals upon PLC activation, we evaluated the involvement of InsP_3_R, which provides the main pathway for ER Ca^2+^ release in fibroblasts (Horie et al., 2014; Berra-Romani et al., 2020). The results obtained demonstrate that, after incubation for 20 min with 2-aminoethoxydiphenyl borate (2-APB, 50 μM), a drug widely used as InsP_3_R inhibitor (Guzmán-Silva et al., 2015), the amplitude of histamine-evoked initial Ca^2+^ transient is significantly reduced compared to untreated cells, both in the presence (Figures 6A,C) and in the absence of extracellular Ca^2+^ (Figures 6B,C). In addition, the number of oscillations was significantly decreased when fibroblasts were preincubated with 2-APB in normal Ca^2+^ (Figures 6A,D) and completely eliminated under 0Ca^2+^ conditions (Figures 6B,D). Even though 2-APB application during histamine-activated Ca^2+^ oscillations caused an immediate and transitory increase in [Ca^2+^]_i_, 2-APB subsequently erased the intracellular Ca^2+^ oscillations and plateau phase; this effect was reversible (Figure 6E). RyR could support InsP_3_-induced intracellular Ca^2+^ oscillations through CICR, as reported in other cell types (Paltauf-Doburzynska et al., 2000). Nevertheless, the acute addition of caffeine (10 mM) mimicked the inhibitory effect of 2-APB by reversibly interrupting the oscillatory Ca^2+^ train (Supplementary Figure S1) in 92.3% of tested cells (60 out of 65 cells). This observation confirms that RyR, which are directly gated by caffeine (Pulina et al., 2010), do not contribute to histamine-induced intracellular Ca^2+^ waves in WI-38 fibroblasts and is consistent with the well-known phenomenon of InsP_3_R inhibition by caffeine (Parker and Ivorra, 1991; Moccia et al., 2003). Taken together, these results suggest a strong involvement of InsP_3_R in histamine-evoked Ca^2+^ transients in lung fibroblasts of the WI-38 cell line.

### Extracellular Ca^2+^ influx contributes to histamine-induced intracellular Ca^2+^ signaling in WI-38 human lung fibroblasts

**FIGURE 7:**
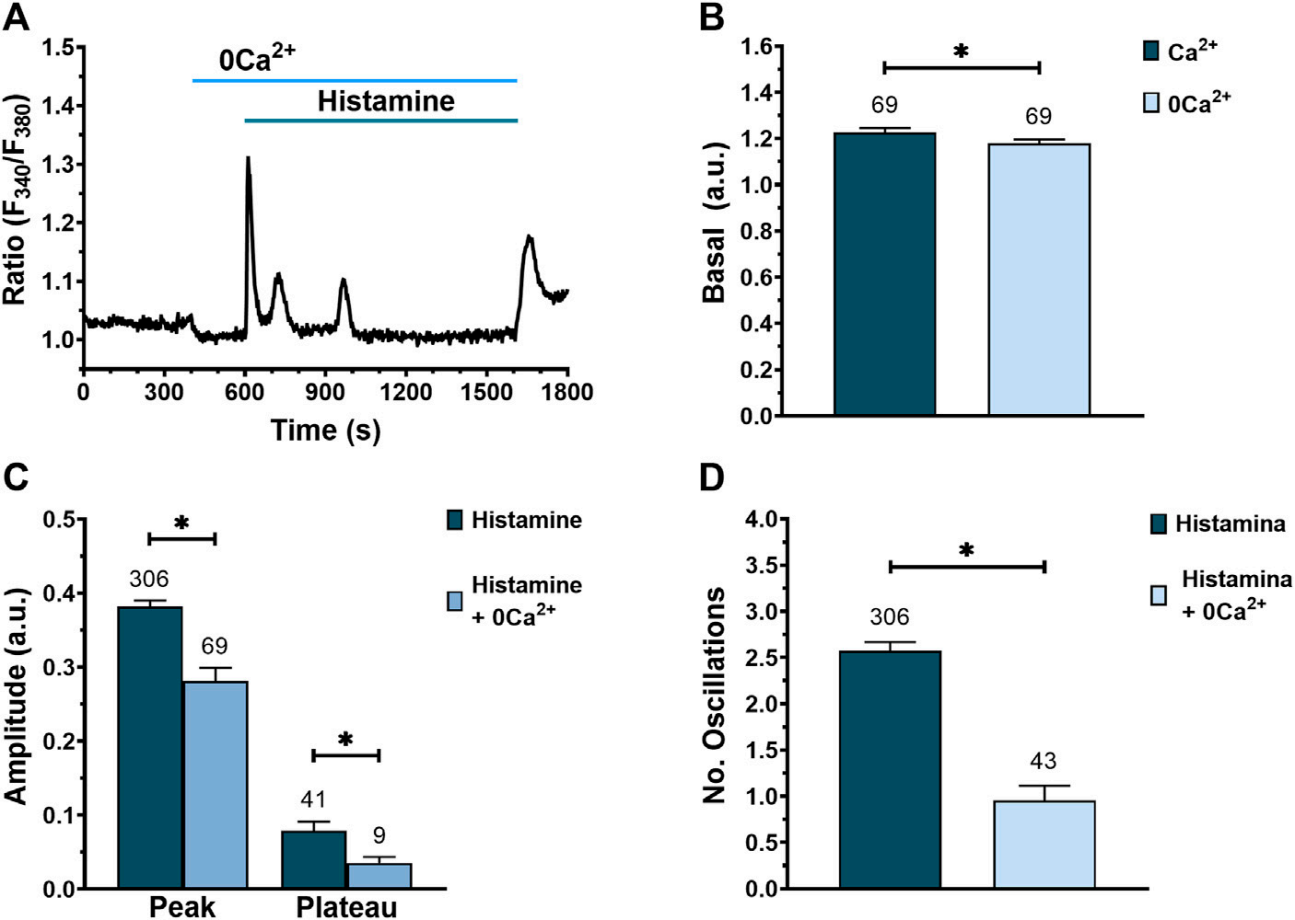
Effect of extracellular Ca^2+^ removal on histamine-evoked Ca^2+^ signal in WI-38 human lung fibroblasts. **(A)** Typical recording of histamine-evoked Ca^2+^ signals in the absence of extracellular Ca^2+^ (0Ca^2+^). **(B)** Mean ± SE of basal [Ca^2+^]_i_ in fibroblasts exposed to an extracellular solution with (dark green bar) or without extracellular Ca^2+^ (blue bar). Comparison between groups was performed using the t-Student test (* = *p* ≤ 0.05). **(C)** Mean ± SE of peak and plateau amplitudes of the Ca^2+^ signal evoked by histamine in fibroblasts expose to an extracellular solution with (dark green bar) or without extracellular Ca^2+^ (blue bar). Comparison between groups was performed using the *t*-Student test for peak amplitude data and Mann-Whitney test for plateau amplitude data (* = *p* ≤ 0.05). **(D)** Mean ± SE of the number of oscillations recorded during the first 400 s after histamine application in an extracellular solution with (dark green bar) or without extracellular Ca^2+^ (blue bar). Comparison between groups was performed using the Mann-Whitney test (* = *p* ≤ 0.05). The numbers in the figures represents the number of cells studied.

Next, we evaluated the contribution of extracellular Ca^2+^ to histamine-evoked Ca^2+^ signals by exposing the WI-38 fibroblasts to histamine in the absence of external Ca^2+^ to prevent Ca^2+^ entry across the plasma membrane (Berra-Romani et al., 2020). Histamine elicited an immediate increase in [Ca^2+^]_i_ in the absence of extracellular Ca^2+^ in 66 of 69 cells (Figure 7A). Of note, exposure of WI-38 cells to 0Ca^2+^ conditions caused a significative reduction in basal [Ca^2+^]_i_ (Figures 7A,B), which is consistent with the presence of a constitutive Ca^2+^ entry pathway (Zuccolo et al., 2018). When histamine (300 μM) was applied to fibroblasts under 0Ca^2+^ conditions, the peak and plateau amplitudes (Figure 7C), as well as the number of intracellular Ca^2+^ oscillations (Figure 7D), were significantly reduced compared to control conditions. In particular, Ca^2+^ oscillations rapidly run down in the absence of Ca^2+^ entry (Figure 7A). Furthermore, ongoing Ca^2+^ oscillations reversibly ceased upon removal of extracellular Ca^2+^ (Supplementary Figure S2) in 97.1% of tested cells (66 out of 68 cells). These results indicate that the peak and plateau amplitude as well the number of Ca^2+^ oscillations evoked by histamine are due to both Ca^2+^ release from the ER and Ca^2+^ influx from the extracellular medium.

### Blocking Ca^2+^ entry through VOCs does not affect histamine-evoked intracellular Ca^2+^ signals

**FIGURE 8:**
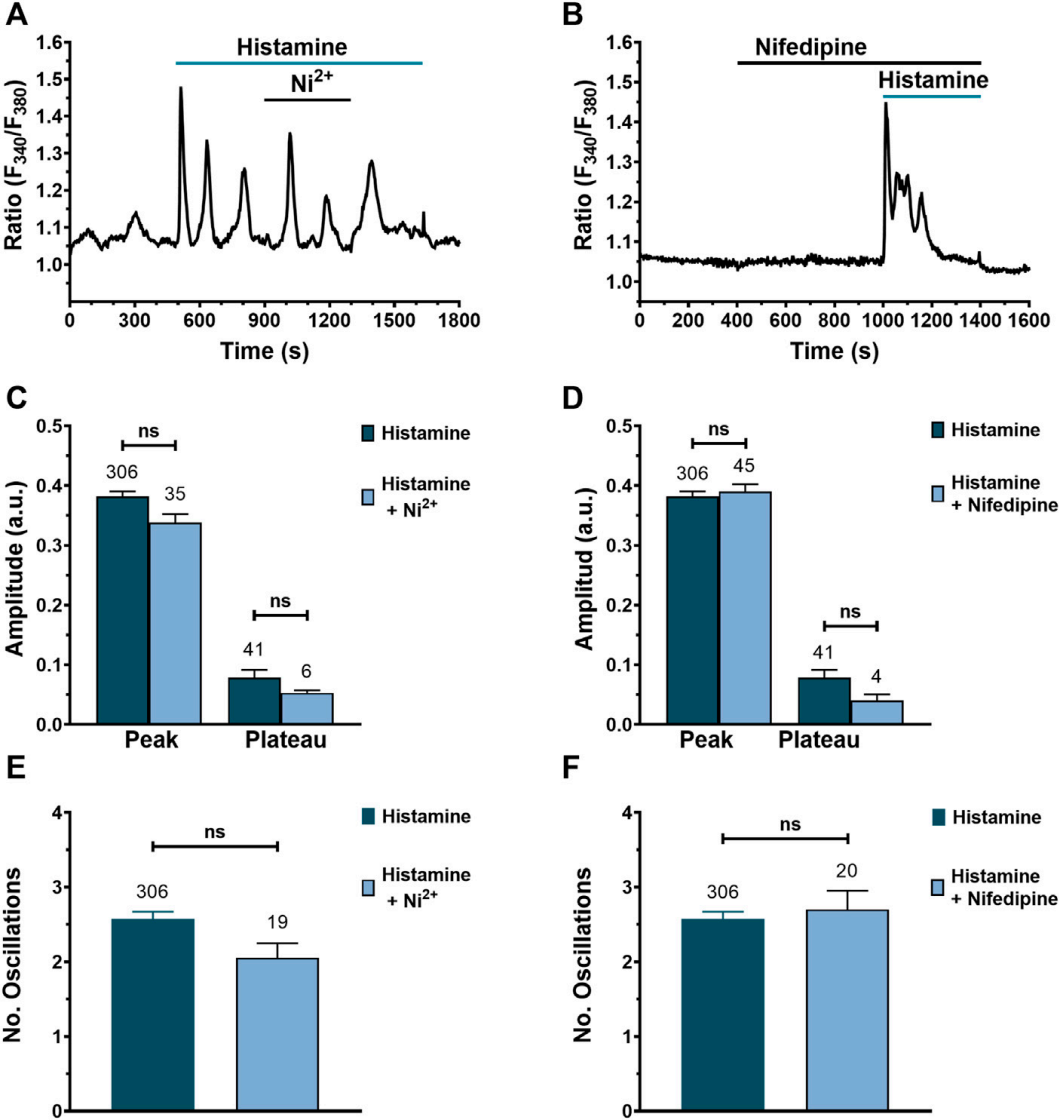
Blocking Ca^2+^ entry through VOCs does not affect histamine-evoked intracellular Ca^2+^ signals in WI-38 human lung fibroblasts. **(A)** Typical recording of histamine-evoked Ca^2+^ signals in the presence of nickel (10 μM). **(B)** Typical recording of histamine-evoked Ca^2+^ signals in the presence of nifedipine (10 μM). Comparison between groups was performed using the Mann-Whitney test (ns = not statistics differences between groups). **(C)** Mean ± SE of the peak and plateau amplitudes of the Ca^2+^ response to histamine (300 μM) in absence (dark green bar) or presence of the non-specific VOC inhibitor, nickel 10 μM (blue bar). **(D)** Mean ± SE of the peak and plateau amplitudes of the Ca^2+^ response to histamine (300 μM) in absence (dark green bar) or presence of the specific VOC inhibitor, nifedipine 10 μM (blue bar). **(E)** Mean ± SE of the number of oscillations recorded during the first 400 s after histamine application in presence (dark green bar) or presence of the unspecific VOC inhibitor, nickel 10 μM (blue bar). **(F)** Mean ± SE of the number of oscillations recorded during the first 400 s after histamine application in presence (dark green bar) or absence of the specific VOC inhibitor, nifedipine 10 μM (blue bar).

There is evidence for a key role of L-type VOCs in TGF-β-induced Ca^2+^ signaling in human lung fibroblasts (Mukherjee et al., 2015). We, therefore, evaluated their involvement in histamine-induced intracellular Ca^2+^ signaling in WI-38 cells. We exploited a non-specific L-type VOC antagonist (nickel, 10 μM) (Figures 8A,C,E) and a specific VOC antagonist (nifedipine, 10 μM) (Figures 8B,D,F). The results show that there were no significant differences in the peak and plateau amplitudes, as well as in the number of oscillations, when Ca^2+^ entry through VOCs was inhibited with either nickel (Hobai et al., 2000) or nifedipine (Zhang et al., 2007), respectively.

### Ca^2+^ influx through SOCs plays an important role in the plateau and oscillations of histamine-evoked Ca^2+^ transients in WI-38 lung fibroblasts

**FIGURE 9:**
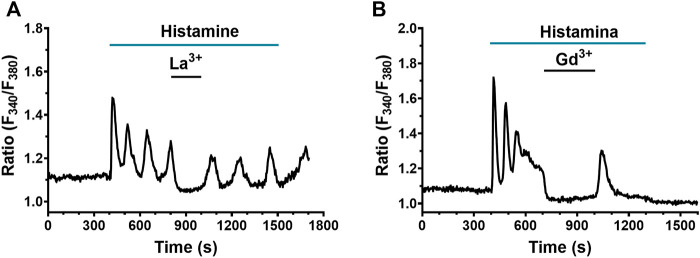
Effect of SOCE blockade on histamine-evoked Ca^2+^ signals in WI-38 human lung fibroblasts. **(A)** Typical recording of histamine-evoked Ca^2+^ signals and effect of the application of 10 µM La^3+^ (*n* = 79). **(B)** Typical recording of histamine-evoked Ca^2+^ signals and effect of the application of 10 μM Gd^3+^ (*n* = 40).

A recent investigation hinted at SOCE as the main Ca^2+^ entry pathway sustaining the Ca^2+^ response to chemical stimuli in human lung fibroblasts (Guzmán-Silva et al., 2015). Therefore, we evaluated the role of SOCs in the Ca^2+^ signal evoked by histamine in WI-38 fibroblasts. The pyrazole-derivative, BTP-2, which is widely employed to inhibit SOCE in non-excitable cells (Prakriya and Lewis, 2015; Moccia et al., 2016; Zhang et al., 2020), increases [Ca^2+^]_i_ in human lung fibroblasts (Guzmán-Silva et al., 2015) and cannot be reliably used to assess SOCE involvement in histamine-evoked extracellular Ca^2+^ entry. In accord, Supplementary Figure S3 shows that BTP-2 (20 µM) induced an immediate elevation in [Ca^2+^]_i_ upon application during the decay phase of the initial Ca^2+^ response to histamine. However, low micromolar doses of the trivalent cations, La^3+^ and Gd^3+^, can also selectively inhibit Orai channels, which provides the main pore-forming subunits of SOCs in both excitable and non-excitable cells (Prakriya and Lewis, 2015; Moccia et al., 2016; Zhang et al., 2020), including human lung fibroblasts (Guzmán-Silva et al., 2015; Vazquez-de-Lara et al., 2018). The results showed that the application of both La^3+^ (10 μM) and Gd^3+^ (10 μM) abrogated the plateau and Ca^2+^ oscillations in 79 and 40 cells exposed to La^3+^ and Gd^3+^, respectively (Figure 9A,B). This effect was reversible upon removal of La^3+^ or Gd^3+^. Intriguingly, the acute addition of La^3+^ and Gd^3+^ also caused a decrease in resting Fura-2 fluorescence ratio below the baseline, which suggests that Orai channels are involved in constitutive Ca^2+^ entry (see Figure 6B). In summary, these results suggest a strong involvement of Ca^2+^ entry through SOCs in the histamine-evoked Ca^2+^ transient in lung fibroblasts of the WI-38 cell line.

### Preliminary characterization of the Ca^2+^ handling machinery in in WI-38 fibroblast

**FIGURE 10:**
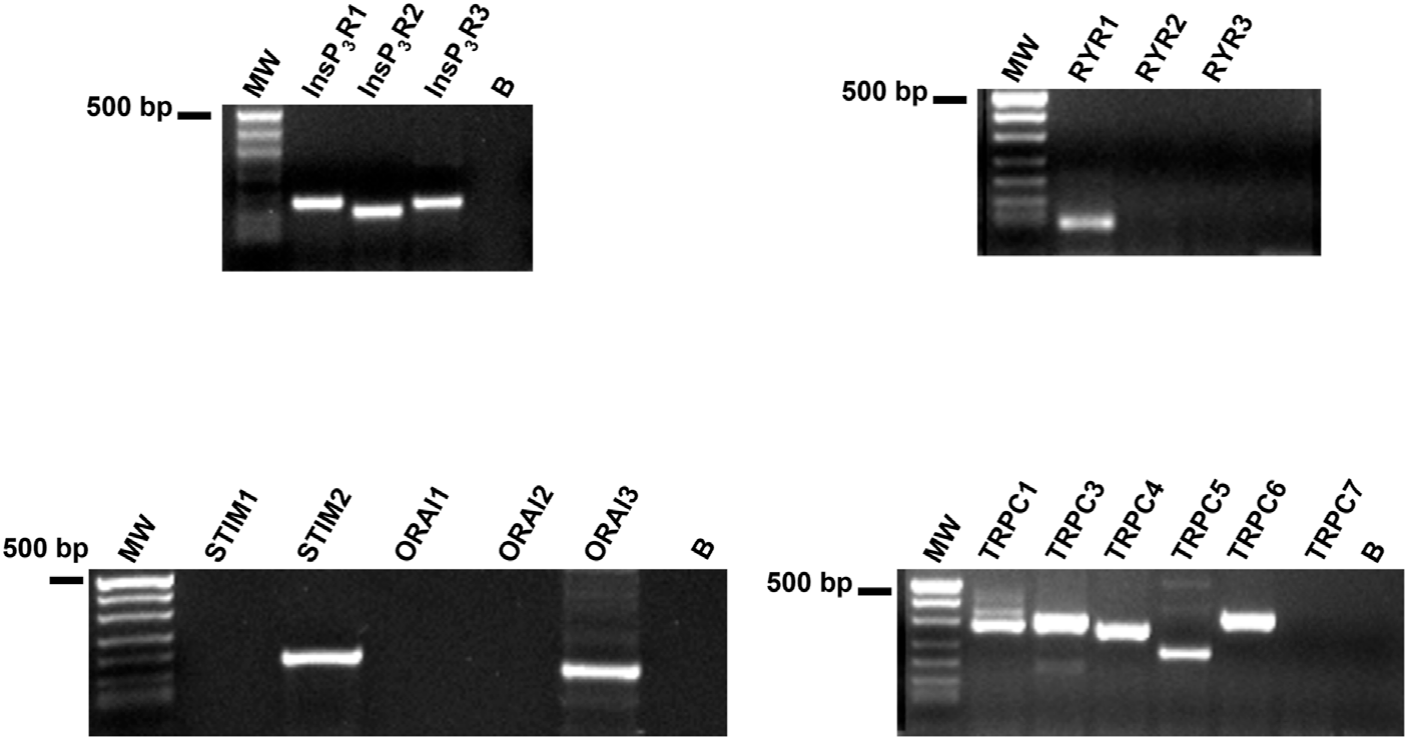
Transcriptomic characterization of the Ca^2+^ handling machinery in WI-38 fibroblasts. Gel electrophoresis of the PCR products are shown. Electrophoresis was performed as indicated in Materials and Methods. The PCR products were of the expected size: InsP_3_R1, 180 bp; InsP_3_R_2_, 158 bp; InsP_3_R_3_, 173 bp; RyR_1_, 75 bp; TRPC1, 307 bp; TRPC3, 336 bp; TRPC4, 300 bp; TRPC5, 221 bp; TRPC6, 341 bp; STIM2, 186 bp; and Orai3, 159 bp. No signal was observed for STIM1 and Orai1. MW, molecular weight markers. B, reaction without the template.

No comprehensive information is available regarding the molecular composition of the Ca^2+^ handling toolkit in WI-38 fibroblasts. Therefore, we performed a preliminary qRT-PCR analysis of the main Ca^2+^-permeable channels that are known to shape the Ca^2+^ response to histamine in other cell types. We used the specific primers described in Table 1, while negative controls were carried out by excluding the reverse transcription reaction, as shown in (Negri et al., 2021). Figure 10 displays that WI-38 lung fibroblasts express the transcripts encoding for all the InsP_3_R isoforms, i.e., InsP_3_R1, InsP_3_R2, and InsP_3_R3, whereas, among the molecular players of the SOCE machinery, only STIM2 and Orai3 paralogues were present. In addition, the mRNAs encoding for RyR1 and most of the members of the Transient Receptor Potential Canonical (TRPC) subfamily, i.e., TRPC1, TRPC3, TRPC4, TRPC5, and TRPC6 (Negri et al., 2019), were also found (Figure 10). Therefore, these findings support the notion that the interaction between InsP_3_R and SOC drive histamine-induced intracellular Ca^2+^ signals in WI-38 human adult lung fibroblasts.

## Discussion

Pulmonary remodeling is the main long-term complication of asthma and occurs as a compensatory response to a persistent inflammatory state of the lower airway epithelium that leads to an irreversible restriction in the airflow, and is a result of a multi-step process known as “scarring.” Mast cells play a crucial pathogenic role in asthma by releasing the autacoid mediator histamine, which stimulates airway smooth muscle cell constriction and hyperplasia, and activates fibroblasts to acquire a contractile phenotype and support pulmonary remodelling. The molecular mechanisms whereby histamine promotes fibroblast proliferation and migration is still unclear, although preliminary evidence indicates that histamine-induced collagen gel contraction is mediated by an increase in [Ca^2+^]_i_ (Horie et al., 2014). Intriguingly, it has long been known that intracellular Ca^2+^ signaling can adopt multiple patterns to orchestrate many of the cellular events that contribute to pulmonary remodeling, including fibroblast proliferation and transformation into myofibroblasts (Janssen et al., 2015). Herein, we provided the first clear-cut characterization of the signaling pathways that shape histamine-induced intracellular Ca^2+^ signals in the WI-38 cell line, which is widely employed to study human lung fibroblasts. Our findings could pave the way towards an alternative strategy to target histamine signaling and thereby dampen airway remodeling in asthma.

Intracellular Ca^2+^ signals regulate a broad spectrum of cellular processes by adopting diverse spatiotemporal dynamics, ranging from single Ca^2+^ transient to repetitive Ca^2+^ transients whose frequency depends on agonist strength (Smedler and Uhlén, 2014). The introduction of high-speed microfluorimetry and high-resolution image analysis techniques have allowed researchers to monitor changes in [Ca^2+^]_i_ in individual cells simultaneously. These techniques have revealed a surprising degree of heterogeneity in the Ca^2+^ responses to the same agonist generated even by cells in the same field of view. Herein, we report that histamine evoked complex Ca^2+^ waveforms in WI-38 fibroblasts that could be classified according to the following patterns: 1) peak, 2) peak and oscillations, 3) peak plateau and oscillations, and 4) peak and plateau. Spatial and temporal heterogeneity of the Ca^2+^ signature can be a hallmark of the Ca^2+^ response evoked by both mechanical (Berra-Romani et al., 2008; Jing et al., 2013) and chemical cues (Dupont and Combettes, 2016; Wacquier et al., 2019). A recent investigation showed that, in normal human lung fibroblasts (NHLF), beractant (a natural surfactant) induced distinct patterns of intracellular Ca^2+^ signals, each comprising an initial Ca^2+^ spike that could be followed either by a) transient, by repetitive Ca^2+^ oscillations, c) a sustained Ca^2+^ plateau or d) a sustained plateau overlapped by repetitive Ca^2+^ oscillations (Guzmán-Silva et al., 2015). Similarly, ATP could elicit either a single Ca^2+^ transient or recurring Ca^2+^ oscillations in NHLF (Janssen et al., 2015), whereas an heterogenous array of agonist-induced intracellular Ca^2+^ signals has been described in fibroblasts deriving from other tissues (Chen et al., 2010; Kemény et al., 2013; Lembong et al., 2015). As expected (Ong et al., 2019), the diverse Ca^2+^ dynamics and the number of oscillations depend on histamine concentration, with the lower concentration being dominated by the peak pattern and the higher concentration being dominated by the peak, plateau and oscillations pattern. In accord, low doses of histamine are predicted to elicit only intracellular Ca^2+^ release, while the Ca^2+^ response to higher doses can also involve extracellular Ca^2+^ entry (Ong et al., 2019). Likewise, a recent investigation showed that histamine was more eager to induce 1 or 2 Ca^2+^ spikes at low picomolar doses, whereas the number and frequency of repetitive Ca^2+^ transients progressively increased with histamine concentration (up to 300 µM) in human microvascular endothelial cells (Berra-Romani et al., 2020). The non-cumulative concentration-response relationship showed that the Ca^2+^ signal evoked by histamine in WI-38 human lung fibroblasts present an *EC_50_* value of 4.96 μM, a maximum concentration of 300 μM, and a threshold concentration of 100 nM. Similarly, histamine induced collagen gel contraction and proliferation in, respectively, primary cultured human lung fibroblasts (Horie et al., 2014) and IMR-90 adult lung fibroblasts (Kunzmann et al., 2007) within a concentration range spanning from 100 nM up to 100 μM. Moreover, the pro-migratory effect of histamine on human lung fetal fibroblasts appeared at a threshold dose of 100 nM (Kohyama et al., 2010). These studies concur with the evidence that H1R mediates histamine-induced proliferation, migration, and collagen gel contraction in human lung fibroblasts. In agreement with these observations, pharmacological manipulation showed that histamine generates a complex increase in [Ca^2+^]_i_ in WI-38 fibroblasts mainly through H1R and, to a lesser extent, through H2R. Furthermore, short-term exposure of the cells to histamine reduced the responsiveness to subsequent applications of the agonist, which is a hallmark of H1R signalling (Smit et al., 1992). Homologous desensitization of H1R has also been reported in smooth muscle preparations (Leurs et al., 1990; Leurs et al., 1991), HeLa cells (Smit et al., 1992), and human gingival fibroblasts (Gutiérrez-Venegas and Rodríguez-Pérez, 2012). Conversely, H3R and H4R do not seem to play a crucial role in the onset of the Ca^2+^ signal. These results were supported by the evidence that histamine-evoked intracellular Ca^2+^ waves were not altered by the PT-dependent ribosylation of the Gα_i/o_ subunit, which triggers the signalling cascades activated downstream of both H3R and H4R (Seifert et al., 2013). Consistent with our observations, Horie et al. (2014) previously reported the involvement of H1R in histamine-induced collagen gel contraction in primary cultured lung fibroblasts, whereas H2R could be responsible for a small Ca^2+^ response occurring in the presence of diphenhydramine, a specific H1R-antihistamine.

The mechanisms that control the mobilization of cytosolic Ca^2+^ are key to the regulation of numerous eukaryotic cell functions (Clapham, 2007). Therefore, after identifying the HR subtype responsible for histamine-evoked Ca^2+^ signaling, we set out to dissect the molecular underpinnings of the Ca^2+^ transient. H1R is a GqPCR that can signal an increase in [Ca^2+^]_i_ by stimulating PLCβ to synthesize InsP_3_ and trigger ER Ca^2+^ release through InsP_3_R (Berra-Romani et al., 2012; Seifert et al., 2013; Berra-Romani et al., 2020). In accord, the Ca^2+^ response to histamine still occurred in the absence of extracellular Ca^2+^, although it rapidly run down after 1-3 Ca^2+^ spikes. Furthermore, histamine-evoked intracellular Ca^2+^ signals were strongly reduced by blocking PLCβ activity with U73122, but not its inactive analog, U73343. Furthermore, the initial Ca^2+^ peak was significantly reduced as compared to control, i.e., untreated, cells upon inhibition of InsP_3_R with 2-APB. This inhibitory effect was observed both in the presence and in the absence of extracellular Ca^2+^. 2-APB significantly decreased the number of oscillations under normal Ca^2+^ conditions, while it completely erased the spiking response under 0Ca^2+^ conditions. Of note, inhibition of InsP_3_Rs with 2-APB, despite decreasing the amplitude of the Ca^2+^ transients, did not completely eliminate histamine-induced Ca^2+^ signals, as previously reported both in fibroblasts (Horie et al., 2014) and in other cell types (Berra-Romani et al., 2020). Unlike histamine, the Ca^2+^ response to beractant in WI-38 fibroblasts was fully abrogated by 2-APB (Guzmán-Silva et al., 2015). This discrepancy can be explained by invoking several hypotheses. First, the ability of 2-APB to penetrate the cell membrane may significantly vary among different cell types: this feature could explain the high sensitivity to 2-APB observed in some cells, but not in others (Soulsby and Wojcikiewicz, 2002). Second, the degree of 2-APB-dependent inhibition could depend on the histamine concentration employed to characterize the Ca^2+^ response in WI-38 lung fibroblasts. For instance, early work carried out in HeLa cells showed that 100 µM 2-APB was able to completely inhibit the ATP-evoked Ca^2+^ response at all the tested concentrations, while histamine-evoked Ca^2+^ signals were only slightly reduced at high agonist doses (i.e., 100 µM) (Peppiatt et al., 2003). It is likely that a higher concentration of 2-APB is required to block InsP_3_R recruited by histamine in WI-38 lung fibroblasts. However, we did not increase 2-APB concentration to avoid the concentration-dependent side-effects that have been associated to this powerful InsP_3_R inhibitor, such as SOCE inhibition and SERCA modulation (Gambardella et al., 2021). The primary role of InsP_3_R in the Ca^2+^ response to histamine was further corroborated by caffeine, which reversibly inhibited, rather than enhancing, histamine-induced intracellular Ca^2+^ oscillations. At the concentration employed in the present investigation, caffeine can either stimulate RyR (Pulina et al., 2010) or inhibit InsP_3_R (Parker and Ivorra, 1991; Moccia et al., 2003). Therefore, the blocking effect of caffeine further confirms that InsP_3_R are the main responsible for the rhythmical ER Ca^2+^ release induced by caffeine in WI-38 fibroblasts.

While the complex increase in [Ca^2+^]_i_ is triggered by ER Ca^2+^ mobilization through InsP_3_R, the Ca^2+^ response is maintained over time by extracellular Ca^2+^ entry. In accord, removal of external Ca^2+^ resulted in the decrease of the Ca^2+^ peak and Ca^2+^ plateau amplitudes, and in the number of Ca^2+^ oscillations evoked by histamine. These findings concur with previous studies showing that Ca^2+^ influx through the plasma membrane sustains intracellular Ca^2+^ oscillations induced by H1R stimulation in several cell types, including cerebrovascular endothelial cells (Berra-Romani et al., 2020), HeLa cells (Sauvé et al., 1991), and vascular smooth muscle cells (Espinosa-Tanguma et al., 2011). SOCE is activated upon depletion of the ER Ca^2+^ pool and represents the Ca^2+^ entry pathway that sustains the Ca^2+^ signal induced by agonists stimulation in fibroblasts from different tissues, including human mammary gland (Sadras et al., 2021b), human heart (Chung et al., 2021), human skin (Wu et al., 2019), and human lungs (Guzmán-Silva et al., 2015). It has been nicely documented that low (1–10) micromolar doses of the trivalent cations, La^3+^ and Gd^3+^, plug the access to the Orai channel inner pore, thereby specifically inhibiting SOCE (Prakriya and Lewis, 2015; Moccia et al., 2016; Zhang et al., 2020). In accord, the application of either SOC blocker at 400 s after histamine application completely abolished the oscillations and suppressed the Ca^2+^ plateau in WI-38 lung fibroblasts. Similar results were achieved by the acute addition of 10 µM La^3+^ and 10 µM Gd^3+^ on the long-lasting elevation in [Ca^2+^]_i_ evoked in the same cells by beractant (Guzmán-Silva et al., 2015). VOC represent an alternative pathway for extracellular Ca^2+^ entry in NHLF, as shown for the intracellular Ca^2+^ oscillations induced by transforming growth factor β (Mukherjee et al., 2015). However, two structurally unrelated VOC inhibitors, i.e., nickel and nifedipine, did not affect histamine-evoked intracellular Ca^2+^ signals in WI-38 cells. We hypothesize that histamine-dependent SOC activation does not depolarize the membrane potential to such an extent to induce VOC activation.

A preliminary qRT-PCR analysis of the Ca^2+^ handling machinery confirmed that WI-38 fibroblasts express all the three known InsP_3_R isoform and two of the molecular components required to activate SOCE, i.e., STIM2 and Orai3. These data are therefore consistent with the results provided by the pharmacological manipulation of the Ca^2+^ response. Conversely, all Orai and STIM isoforms were detected in human cardiac fibroblasts (Cendula et al., 2021), in which they support spontaneous Ca^2+^ oscillations (Chen et al., 2010). Intriguingly, Orai3 can be directly activated by 50 µM 2-APB independent from ER Ca^2+^ store depletion (Zhang et al., 2020), which might explain the immediate rise in [Ca^2+^]_i_ that occurs upon 2-APB application in the presence (see Figure 6F) but not in the absence (not shown) of extracellular Ca^2+^. RyR1 transcript was also found, but it is unlikely to contribute to histamine-evoked intracellular Ca^2+^ signals, as shown by the inhibitory effect of caffeine. WI-38 fibroblasts also express the transcripts encoding for TRPC1-TRPC6, but the pharmacological sensitivity of histamine-evoked Ca^2+^ entry to 10 µM La^3+^ and Gd^3+^ argues against the involvement of TRPC isoforms. Moreover, a recent investigation demonstrated that TRPC channels do not support SOCE in primary murine lung fibroblasts (Bendiks et al., 2020). Conversely, TRPC channels, which present a single-channel conductance that is 1000-fold larger than Orai3, could be activated by transforming growth factor β and thereby lead to VOC activation via strong membrane depolarization (Mukherjee et al., 2015). Quite surprisingly, this was the first molecular characterization of the Ca^2+^ toolkit in human pulmonary fibroblasts. The selective expression of STIM2 as ER Ca^2+^ sensor might explain the rapid fall in resting [Ca^2+^]_i_ observed upon removal of external Ca^2+^. In accord, STIM2 is activated only by a mild depletion of the ER Ca^2+^ store and can drive the activation of a constitutive Ca^2+^ influx (Brandman et al., 2007). In agreement with this hypothesis, preliminary evidence showed that µM La^3+^ and Gd^3+^ reduced the basal Ca^2+^ entry, thereby suggesting that SOC also support the resting Ca^2+^ permeability of WI-38 fibroblasts (Sanchez-Collado et al., 2020). It is worth of pointing out that the most frequent Ca^2+^ patterns evoked by high doses of histamine, i.e., mode 2) peak-oscillations, 64.7%, and mode 3) peak-plateau-oscillations, 13.39%, entail the occurrence of intracellular Ca^2+^ oscillations. In agreement with this observation, STIM2 and Orai3 can enhance the percentage of cells showing intracellular Ca^2+^ oscillations upon GqPCR stimulation (Yoast et al., 2020; Emrich et al., 2021). Furthermore, mathematical modelling has shown that, because of their distinct sensitivity to cytosolic Ca^2+^, InsP_3_R3 may provide a constant release of Ca^2+^ that stimulates InsP_3_R1 and InsP_3_R2 to rhythmically release ER stored Ca^2+^, whereas SOCE maintains the Ca^2+^ response by ensuring ER Ca^2+^ refilling (Dupont and Croisier, 2010; Dupont, 2014). However, the periodic Ca^2+^ transients transition into a sustained plateau either when InsP_3_R3 expression increases (Okumura et al., 2022) or when ER Ca^2+^ release through InsP_3_R3 is enhanced by the tight coupling with the ER-embedded protein, Jaw1 (Okumura et al., 2022). Therefore, the molecular assortment of the distinct STIM/Orai and InsP_3_R isoform, as well as cell-to-cell variability in their expression, subcellular distribution, or posttranslational regulation, could contribute to pattern a heterogenous array of Ca^2+^ signatures in WI-38 adult lung fibroblast (Ishida et al., 2014; Guzmán-Silva et al., 2015; Bartok et al., 2019; Wilson et al., 2020). Conversely, cell cycle asynchrony is an unlikely explanation of the cell-to-cell heterogeneity of histamine-evoked Ca^2+^ waves because our experiments were performed in fibroblasts devoid of serum for 48 h, which causes cell cycle arrest in G0 phase (Santella, 1998). Similarly, previous studies in fibroblasts and other cell types have reported that this variability in the intracellular Ca^2+^ dynamics is not due to cell cycle asynchrony (Ambler et al., 1988; Byron and Villereal, 1989; Dragoni et al., 2011; Guzmán-Silva et al., 2015; Okumura et al., 2022).

In conclusion, the present investigation showed that histamine induces a dose-dependent increase in [Ca^2+^]_i_ in the widely employed human pulmonary fibroblast cell line, WI-38. The Ca^2+^ signal is mainly triggered by H1R and can adopt multiple signatures, the most common of which encompasses intracellular Ca^2+^ oscillations, which have long been known to stimulate gene expression, proliferation, contraction and migration in human pulmonary fibroblasts (Janssen et al., 2015). The Ca^2+^ response to histamine is triggered by ER Ca^2+^ release through InsP_3_R and maintained over time by SOCE activation. These data suggest that the Ca^2+^ handling machinery could provide an alternative molecular target to prevent the pernicious effects of histamine on lung fibroblasts in asthmatic patients, as recently suggested also for pulmonary hypertension (Bikou et al., 2022), *Streptococcus* pneumoniae-induced lung injury (Ali et al., 2022), and asthma itself (Johnson et al., 2022). Much research remains therefore to be done to assess this issue, although the work presented here provides valuable information for understanding the mechanisms that regulate histamine-evoked Ca^2+^ signaling in lung fibroblasts. A limitation of the present study is that we did not use lung fibroblasts from an asthmatic model or from patients with asthma. Future work will have to compare the effect generated by histamine in lung fibroblasts from normal airways and in lung fibroblasts from asthmatic airways.

## Data Availability

The raw data supporting the conclusion of this article will be made available by the authors, without undue reservation.

## Supplementary material

SUPPLEMENTARY FIGURE S1 Effect of caffeine on histamine-evoked Ca^2+^ signals in WI-38 human lung fibroblasts. Typical recording of histamine-evoked Ca^2+^ signals and effect of the application of 10 mM caffeine.

SUPPLEMENTARY FIGURE S2 Effect of extracellular Ca^2+^ removal on histamine-evoked Ca^2+^ oscillations in WI-38 human lung fibroblasts. Typical recording of histamine-evoked Ca^2+^ oscillations and effect of extracellular Ca^2+^ removal.

SUPPLEMENTARY FIGURE S3 Effect of BTP-2 on histamine-evoked Ca^2+^ signals in WI-38 human lung fibroblasts. Typical recording of histamine-evoked Ca^2+^ signals and effect of BTP-2 (*n* = 76).
