# Supplementary figures and images for: Histamine activates an intracellular Ca2+ signal in normal human lung fibroblast WI-38 cells
Source: Front Cell Dev Biol. 2022 Sep 2;10:991659. doi: 10.3389/fcell.2022.991659 (PMC9478493; doi:10.3389/fcell.2022.991659)

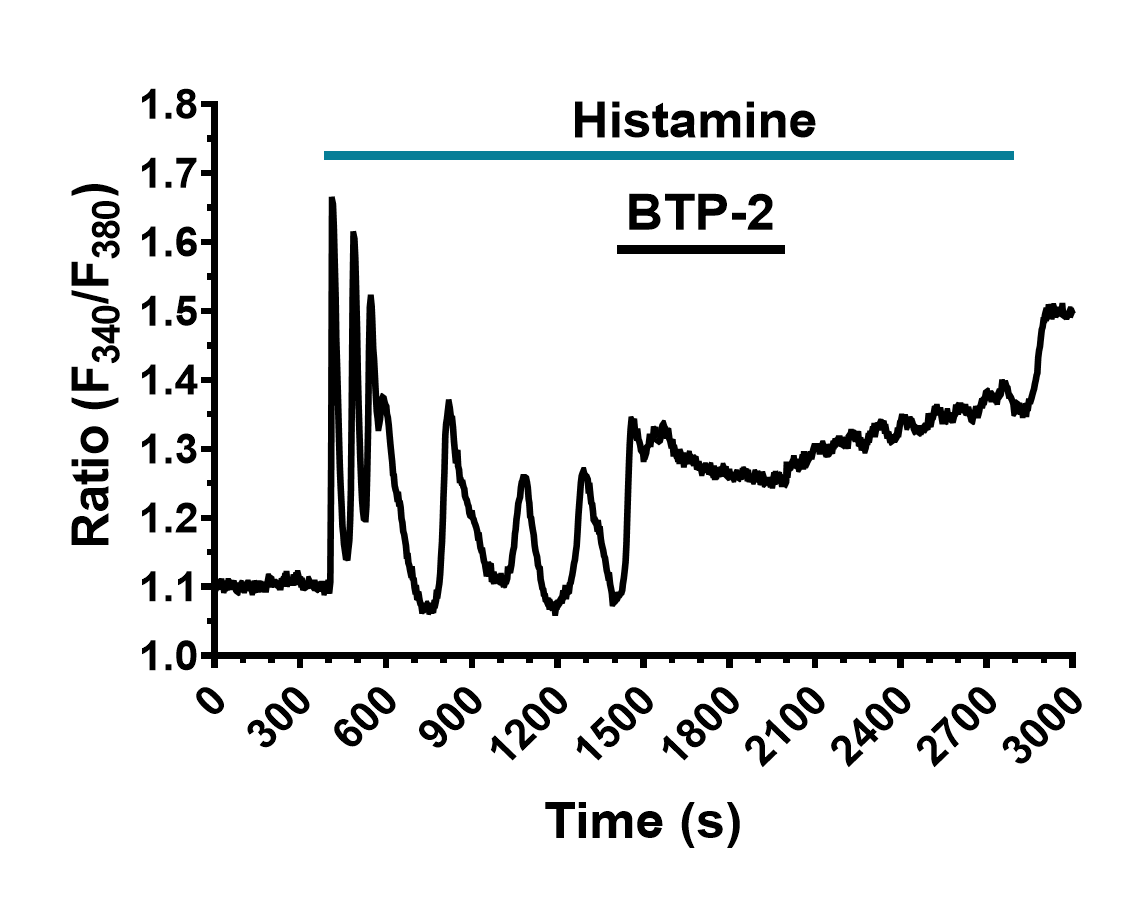

Supplement: Supplementary file 1 [file Image3.TIF]

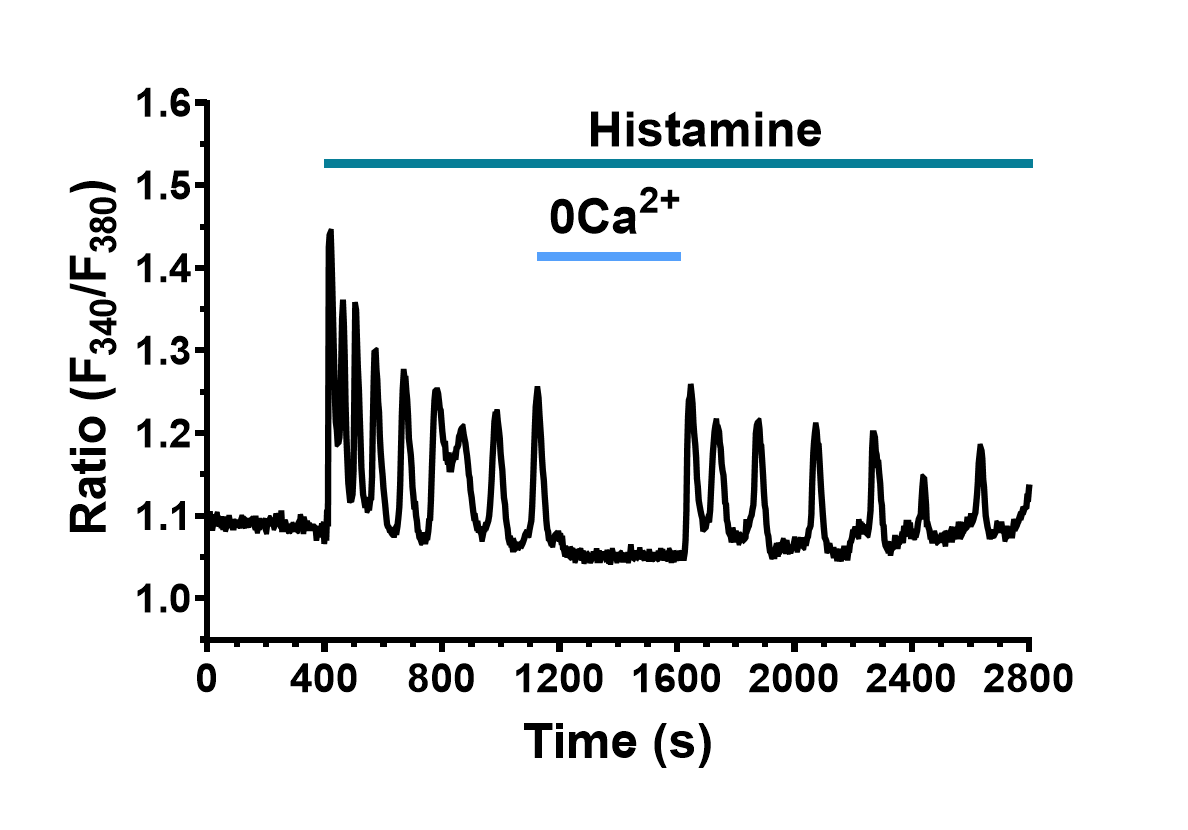

Supplement: Supplementary file 2 [file Image2.TIF]

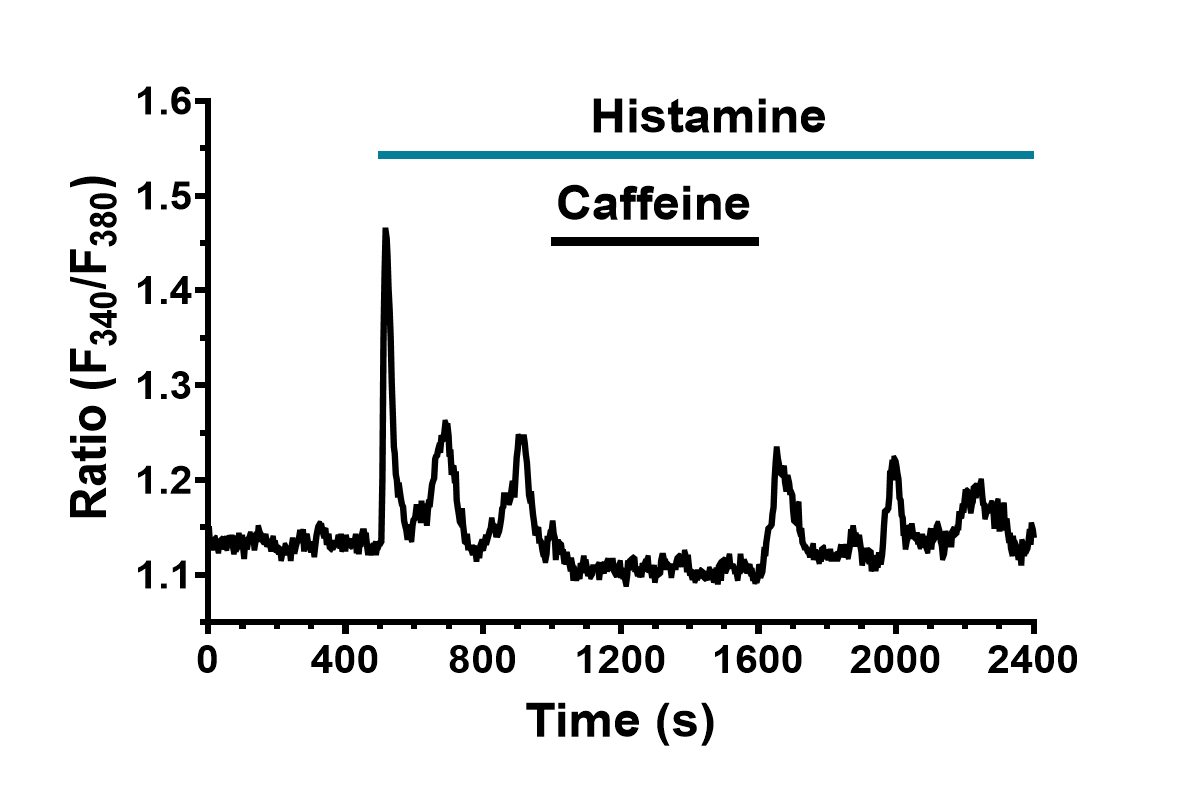

Supplement: Supplementary file 3 [file Image1.TIF]
